# 3D Printability Assessment of Poly(octamethylene maleate (anhydride) citrate) and Poly(ethylene glycol) Diacrylate Copolymers for Biomedical Applications

**DOI:** 10.1021/acsapm.2c00531

**Published:** 2022-07-07

**Authors:** Dominic J. Wales, Meysam Keshavarz, Carmel Howe, Eric Yeatman

**Affiliations:** † Hamlyn Centre, Institute of Global Health Innovation, Imperial College London, London SW7 2AZ, U.K.; ‡ Department of Bioengineering, Imperial College London, London SW7 2AZ, United Kingdom; § Department of Electrical and Electronic Engineering, Imperial College London, London SW7 2AZ, U.K.

**Keywords:** 3D printing, biomaterial, tunable properties, copolymers, bioadhesion, POMaC

## Abstract

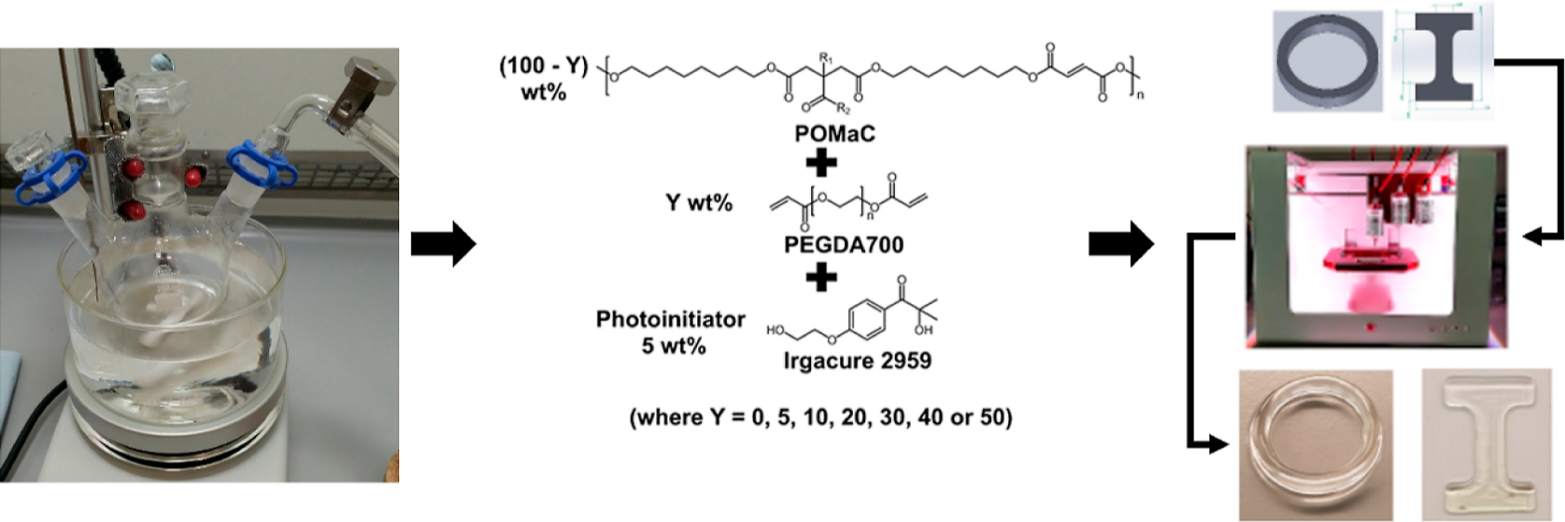

Herein, we present the first example of 3D printing with poly(octamethylene maleate (anhydride) citrate) (POMaC), a bio-adhesive material which has shown particular promise for implantable biomedical devices. The current methods to fabricate such devices made from POMaC are hindered by the imposed constraints of designing complex molds. We demonstrate the feasibility of exploiting additive manufacturing to 3D print structural functional materials consisting of POMaC. We present 3D printing of biomaterial copolymers consisting of mixtures of poly(ethylene glycol) diacrylate (PEGDA) and POMaC at different ratios. The required parameters were optimized, and characterization of the printing fidelity and physical properties was performed. We have also demonstrated that a range of mechanical properties can be achieved by tuning the POMaC/PEGDA ratio. The biocompatibility of the copolymers was ascertained via a cell viability assay. Such tunable 3D printed biomaterials consisting of POMaC and PEGDA will have significant potential application in the development of functional biomaterial tissue scaffolds and biomedical devices for the future of personalized medicine.

## Introduction

**Figure 1:**
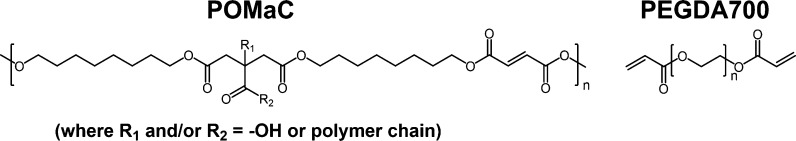
Structures of the polymers POMaC and PEGDA700.

Citric acid-based elastomers (CABEs) are a class of biodegradable elastomers, the structures of which are based around the multifunctional citrate monomer.^1^ The citrate moiety can participate in ester cross-link formation between the elastomer chains, can form hydrogen bonding interactions, and can also facilitate bioconjugation for tuning of the material properties.^1^ Such versatility, combined with the non-toxic nature of citric acid, has led to a significant growth in research attention focused on the use of CABEs for biomedical applications. Since the early work on CABEs by Yang et al. in 2004,^2,3^ there have been many examples of biomedical materials and devices fabricated from citrate-based biomaterials.^3−18^ In particular, a type of CABEs that exhibit exceptional tunable multifunctionality are poly(alkylene maleate citrates). These polymers consist of alkylene, maleate, (introducing a polymerizable alkene group into the backbone of the polymer) and citrate moieties and can be cross-linked to form thermoset elastomers, either by one or both of the following two mechanisms: thermal cross-linking via ester formation and/or photocross-linking via the alkene moieties. One poly(alkylene maleate citrate) that has received much research attention is poly(octamethylene maleate (anhydride) citrate) citrate (POMaC) (Figure 1). Formed from a co-condensation reaction of citric acid, 1,8-octanediol, and maleic anhydride, it has been demonstrated that a high degree of control over the desired mechanical, adhesive, and biodegradability properties of the resulting cross-linked thermoset elastomer is possible, by tuning the amount of both thermal and/or photocross-linking.^8^ Undeniably, this high degree of tunability means that POMaC is well suited for use as a scaffold for minimally invasive delivery of functional tissues and for the development of biodegradable sticky “AngioChip” patches for direct surgical anastomosis, both of which have been demonstrated in animal models.^6,17^ Currently, thermoset POMaC elastomer-based devices are fabricated using molding processes, however, this methodology is more suited for scaled-up fabrication due to the high cost of the design and subsequent fabrication of the mold and there are constraints in achieving complex geometries.^3^ On the other hand, 3D printing offers rapid and facile fabrication of intricate structures in smaller volumes, which is highly desirable when making bespoke biomedical devices for personalized medical applications.^19^ Therefore, 3D printing has been widely utilized to print advanced functional materials^20−26^ and biomedical implants or devices.^27−30^ 3D bioprinting also allows for the fabrication of structures containing living cells within biomaterial matrices.^31−34^ To this end, there is a need for 3D printing of POMaC. However, to date there are no examples of 3D printed POMaC, and only a few examples in the literature of 3D printing of the wider range of CABEs, some of which use sacrificial co-printed mold materials.^35−41^ Direct ink writing of CABEs can be achieved through the use of filler materials, such as chitin nanocrystals, as this increases the viscosity of the CABE ink to achieve the desired printing resolution; however, filler material loadings of up to 40 weight percentage (wt %) are required which can affect the preferred mechanical and physiochemical properties.^40^ Specifically, for POMaC, the cross-linking time to achieve gelation is on the order of several minutes, which makes the use of POMaC in 3D printing impractical, without changing the chemical structure of the elastomer as reported by Savoji et al.^39^ If this cross-linking time was decreased sufficiently, POMaC could be a desirable material for 3D printing of biofunctional materials. As a solution, we have investigated the use of poly(ethylene glycol) diacrylate (*M*_n_ = 700) (PEGDA700, Figure 1) as a copolymer cross-linker for direct ink writing of POMaC-based materials. The reasoning for using PEGDA as a copolymer cross-linker is fourfold: i) it increases the concentration of available double bonds to undergo radical polymerization, thus decreasing the time needed to achieve sufficient cross-linking for solidification in the timescale for extrusion 3D printing; (ii) the polymerization time of PEGDA when photocross-linked in the presence of ≥2 wt % of photoinitiator is on the order of seconds;^42^ (iii) PEGDA has been demonstrated as a suitable copolymer for fabrication of materials with tunable physical properties;^43−45^ and (iv) the resulting PEG-based polymer formed from polymerization of PEGDA is bioinert.^46,47^

Herein, 3D printing of a copolymer biomaterial with different ratios of POMaC and PEGDA700 (POMaC/PEGDA) is explored. PEGDA700 was chosen as it is a liquid at room temperature and pressure, unlike commercially available PEGDA polymers with molecular weights ≥1000 Da which are solids. Furthermore, compared to other PEGDA polymer that are liquid at room temperature and pressure, PEGDA700 has been shown to demonstrate the fastest polymerization rates.^48^ The optimal parameters for (extrusion-based) 3D printing of the copolymer biomaterial were determined, as well as analysis of the printing fidelity and elucidation of the achievable range of mechanical and physical properties through tuning the POMaC/PEGDA ratio. The 3D printed copolymer biomaterials were determined to be biocompatible by a cell viability assay. To the best of our knowledge, this is the first example of 3D printing of POMaC with tunable physical/mechanical attributions, and this work is a systematic study into 3D printing of POMaC-based copolymer biomaterials which allows for POMaC to be used in future biomedical applications.

## Experimental Section

### General Experimental Procedure

**Figure 2:**
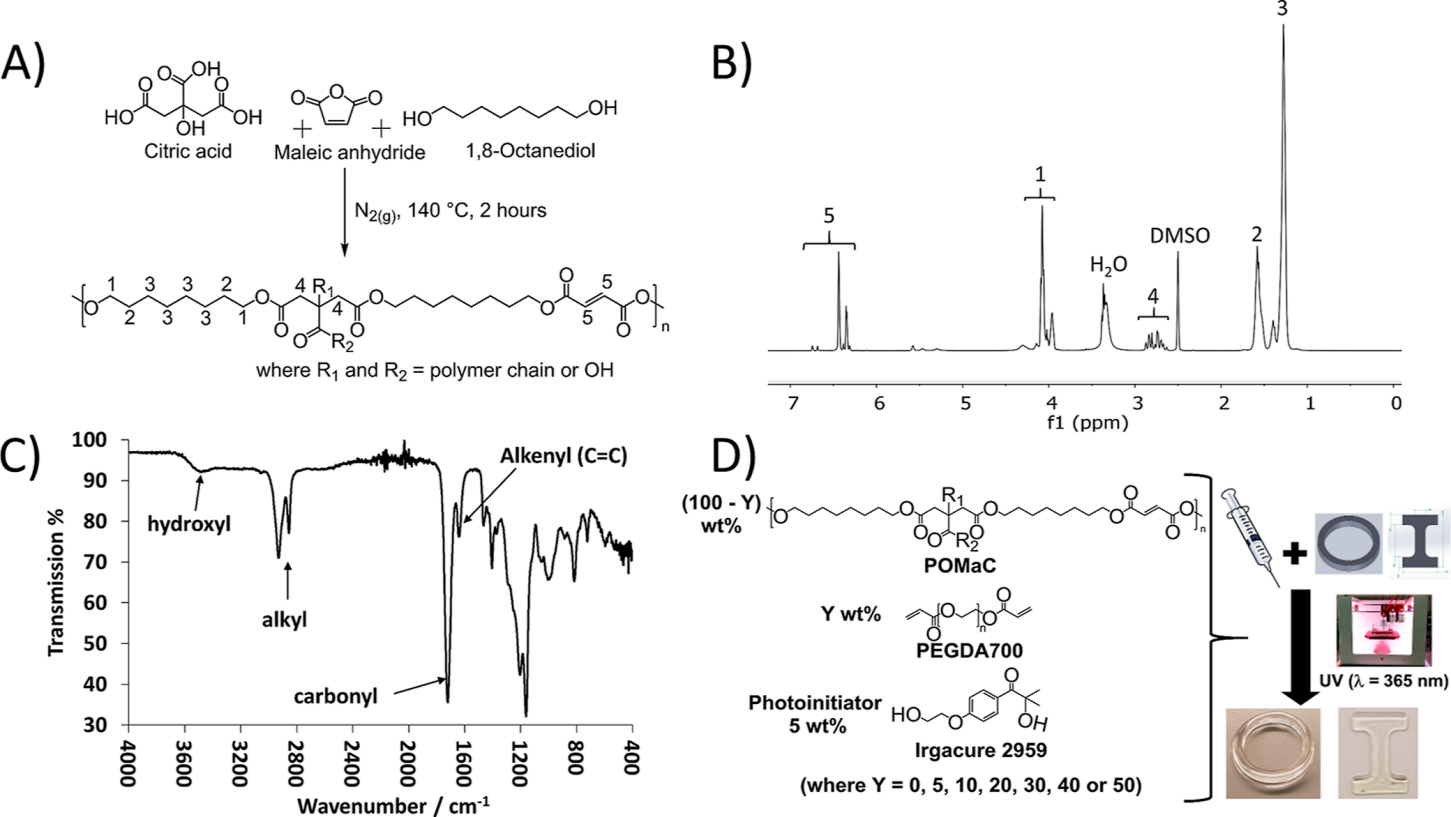
(A) POMAC pre-polymer was synthesized through a co-condensation reaction of the three different monomers (citric acid, maleic anhydride, and 1,8-octanediol) in a 1:4:5 M ratio at 140 °C for 2 h under a nitrogen atmosphere. (B) ^1^H-NMR spectrum of the POMaC pre-polymer confirming the structure with the structural assignments as defined in (A). (C) FT-IR spectrum of the POMaC pre-polymer highlighting the key spectral features of the key functional group moieties. (D) POMaC-containing inks, consisting of POMaC pre-polymer (100–*Y*) wt % combined with *Y* wt % PEGDA700 (where *Y* = 0, 5, 10, 20, 30, 40, or 50) and 5 wt % Irgacure 2959 photoinitiator, were 3D printed or molded (not depicted) using the UV LED (λ = 365 nm) curing system on the 3D printer.

Unless otherwise stated, all reagents were purchased from Merck, TCI, Acros Organics, and Fisher Scientific and used without additional purification. HiPerSolv CHROMANORM HPLC grade water from VWR was used in all purification experimental steps. Nitrogen gas refers to oxygen free nitrogen, 99.9% purity from BOC (Guildford, UK). FT-IR spectra were collected on a Thermo Scientific Nicolet iS50 FT-IR spectrometer fitted with a diamond ATR module, 4000–400 cm^–1^, 64 scans, and 0.5 cm^–1^ resolution. ^1^H NMR spectra were recorded on a JEOL 400YH (400 MHz) spectrometer at 25 °C, using DMSO-*d*_6_ NMR solvent, 96 scans, and a concentration of POMaC of approx. 5–10 mg.mL^–1^. MALDI mass spectrometry was performed in positive ionization mode using a Waters Micromass MALDI-ToF mass spectrometer and α-cyano-4-hydroxy-cinnamic acid as the matrix. The 3D printer used in this work was an Allevi 3 (Allevi, Philadelphia, USA) equipped with a UV LED (λ = 365 nm, 20 mW.cm^–2^ at 100% intensity) for photopolymerization. The photoinitiator used was Irgacure 2959 (2-hydroxy-4′-(2-hydroxyethoxy)-2-methylpropiophenone), the chemical structure of which is shown in Figure 2D. Irgacure 2959 is a commonly used photoinitiator for 3D printing of hydrogels and biopolymers.^49^ The optimized printing conditions are described in the Results and Discussion section. Thermogravimetric analysis (TGA) was performed using a Mettler Toledo TGA/DSC 1 instrument and the samples were heated in air up to 800 °C with a heating rate of 5 °C·min^–1^. Tensile stress–strain and adhesive testing experiments were performed using a Sauter FH 5 Force Gauge (Max. = 5 N, resolution = 0.001 N).

### Synthesis of POMaC Pre-polymer

POMaC pre-polymer was synthesized using a modified version of a synthesis procedure reported by Tran et al.^8^ In the work reported herein, the reaction time for POMaC was reduced from 3 to 2 h, as it was determined that the POMaC pre-polymer synthesized for 3 h led to a liquid which was qualitatively too viscous for direct ink writing. The synthesis process was repeated several times to obtain a large quantity of POMaC pre-polymer for 3D printing optimization experiments. In a typical synthesis, a dry round-bottom flask, containing a magnetic stirrer bar, was heated to 160 °C under a nitrogen purge. Once at temperature, citric acid (3.84 g, 2.00 × 10^–2^ mol), maleic anhydride (7.84 g, 8.00 × 10^–2^ mol), and 1,8-octanediol (14.62 g, 1.00 × 10^–1^ mol) (reagent molar ratio = 1:4:5) were added to the flask in that order. The reagents were then left to stir vigorously at 160 °C until all the reagents were fully melted, forming a homogeneous clear and colorless liquid reaction mixture. At this point, the reaction temperature was lowered to 140 °C and the reagents were left to react for 2 h under a nitrogen gas purge. After 2 h, the reaction mixture was cooled to room temperature under a nitrogen purge. When cooled, the nitrogen purge was stopped and 1,4-dioxane (∼150 mL) was added to the flask, and the product was dissolved with vigorous stirring resulting in a clear and colorless solution. This solution was then added dropwise to vigorously stirred deionized water (5 L) forming a cloudy white precipitate. This mixture was left to stir for at least 15 h, before allowing the cloudy white gel-like hydrophobic precipitate to settle to the bottom of the beaker and then decanting off the partially cloudy supernatant liquid. This purification process was repeated two times before a cloudy white viscous gel-like liquid was obtained. The product was then lyophilized in a FreeZone 4.5 L Console Freeze Dry System (Labconco, USA) for 3 days to obtain POMaC pre-polymer as a clear and viscous liquid. ^1^H NMR (400 MHz, DMSO-*d*_6_): δ/ppm = 6.76–6.29 (m, **5** (maleic moiety alkenyl protons)); 4.20–3.88 (m, **1** (octamethylene −CH_2_ backbone protons)); 2.92–2.61 (m, **4** (citric acid moiety −CH_2_ protons); 1.69–1.47 (m, **2** (octamethylene −CH_2_ backbone protons)); 1.27 (broad s., **3** (octamethylene −CH_2_ backbone protons)). The bold numbers refer to the structural assignments defined in Figure 2A. NMR data are consistent with spectra reported in the literature.^6^

### POMaC/PEGDA Printing Ink Blends

The preparation of the ink blends was performed in the dark, and brown glass vials were used. First, the photoinitiator Irgacure 2959 was weighed out (5, or 20 wt % for pure POMaC) into the vial, then the appropriate wt % of PEGDA700 was added, followed by the weighed addition of the corresponding wt % of POMaC so that the wt % of PEGDA700 and POMaC added up to 100 wt %. The vials of inks were then sonicated in an ultrasonic bath (35 kHz) for varying lengths of time (specific times given in Table 1) until the photoinitiator was fully dissolved and a homogeneous ink was obtained. However, care was taken to maintain the temperature of the water bath below 45 °C during sonication to avoid thermal polymerization. The ink compositions are detailed below in Table 1.

**Table 1:** Compositions of the POMaC/PEGDA Biomaterial Inks

biomaterial ink name	POMaC quantity/wt %	PEGDA700 quantity (Y)/wt %	PI quantity/wt %^a^	approx. sonication time until full dissolution/mins
50/50	50	50	5	30
60/40	60	40	5	30
70/30	70	30	5	60
80/20	80	20	5	60
90/10	90	10	5	120
95/5	95	5	5 (or 20)^b^	180
100/0	100	0	5 (or 20)^c^	180

a PI = photoinitiator. The photoinitiator used was Irgacure 2959.

b To achieve 3D printing of the 95/5 dog bone pieces, 20 wt % PI was necessary for satisfactory printability. For the 3D printing of the 95/5 rings, 5 wt % PI was used.

c To achieve 3D printing of the 100/0 dog bone pieces, 20 wt % PI was necessary for satisfactory printability. For the 3D printing of the 100/0 rings, 5 wt % PI was used.

### 3D Printing Method

For 3D printing, the inks were loaded into syringes (PSYR5, Allevi, Philadelphia, USA) fitted with 25-gauge Luer Lock dispensing tips (12.7 mm length, 0.25 mm inner diameter, 0.51 mm outer diameter, AD725050PK, Adhesive Dispensing Ltd, Milton Keynes, UK). The geometries of the 3D printed pieces were defined using standard 3D design software (SolidWorks, Dassault Systèmes, France) and converted to *.stl* files for printing. The desired geometries of the .*stl* models (a “dog bone” and a ring) are shown in Figure S1. The pieces were then printed using an Allevi 3 bioprinter (Allevi, Philadelphia, USA) equipped with a UV LED, λ = 365 nm, 20 mW·cm^–2^ at 100% intensity (5 cm distance between UV LED and print specimen). The printing temperature was in the range of 35–40 °C and the print bed temperature was ambient room temperature. The printing conditions are given in Table 2 and discussed in detail in the Results and Discussion section. The 3D printed ring pieces were used for tensile stress–strain experiments.

**Table 2:** 3D Printing Parameters for the Different POMaC/PEGDA Biomaterial Ink Compositions for Ring and “Dog Bone” 3D Printed Pieces

			UV intensity and duration	
biomaterial ink composition	extrusion pressure/PSI	print speed/mm·s^–1^	during print	after print
50/50	10	8	15%	N/A
60/40	10	5	15%	N/A
70/30	30	6	0%	100% for 300 s
80/20	45	6	50%	100% for 300 s
90/10	60	8	100%	N/A
95/5^a^	80	4	100% for 1200 s per slice	100% for 1800 s
100/0^b^	90	4	100% for 1200 s per slice	100% for 1800 s

a Printing of 95/5 “dog bone” pieces used these printing parameters, but 20 wt % photoinitiator was required.

b Printing of 100/0 “dog bone” pieces used these printing parameters, but 20 wt % photoinitiator was required.

### Tensile Stress–Strain Experiments

The tensile stress–strain data of the 3D printed ring specimens were measured using a vertical tensile stress–strain experimental setup as shown in Figure S2. 3D printed ring pieces were placed on the hooks so that the ring pieces were not under tension, but were on the limit of tension upon an increase of displacement in the tension force direction (Figure S2A). Displacement in the tension force direction was applied at a constant rate. The experiments were video recorded and at each displacement value (in mm) the adhesive peak load values (in N) were extracted manually from the videos by transcribing the values from the force gauge readout screen until the ring piece was observed to break. The data were processed as per the formulae defined in ISO-37 to determine average values of elongation % at break (*E*_b_/%) and tensile strength at break (TS_b_/MPa) for each ink composition.^50^ To determine the true stress–elongation % curves as per ISO-37, the cross-sectional areas of the ring pieces were measured using optical microscopy and Fiji image analysis software.^51^ Average Young’s Modulus (*E*) values were calculated by averaging the gradients of linear sections of each true stress–elongation % curve for the 3D printed ring pieces. Representative true stress–elongation % curve examples are shown in Figure S2B. The values of *E*, *E*_b_, and TS_b_ are presented as the means ± standard deviation.

### Adhesive Test Experiments

The adhesive strength data of the specimens, which had been formed by molding, were measured by the pull-off tensile adhesion test method using the experimental setup shown in Figure S3A. Cuboid specimens [7.5 mm × 7.5 mm × (3.9 ± 0.8) mm, *W* × *L* × H] were fabricated by molding of the different composition inks in a 12-well silicone chamber (Thistle Scientific, Glasgow, UK) under the UV LED of the Allevi 3 printer for at least 1 h for 50/50 and 60/40, ∼4 h for 70/30 and 80/20, ∼12 h for 90/10 and up to 24 h for 95/5 and 100/0, with the “power” of the UV set to 100% on the software interface, which corresponds to an intensity of 20 mW.cm^–2^.^52^ The adhesive test experiments were recorded using a camera and the adhesive peak force was extracted manually from the videos by transcribing the highest magnitude value registered on the force gauge readout screen before pull-off (due to adhesive failure and not cohesive failure) of the specimen from the stainless-steel dolly was achieved. The experimental method is described schematically in Figure S3B-D. Any specimens that underwent cohesive failure were discarded and the values not recorded.

### Degradation Experiments

The degradation experiments were performed in NaOH solution (1 M). This accelerated method was chosen as the degradation of POMaC and PEGDA in phosphate-buffered saline (PBS) or physiologically similar serum-containing media has been extensively established in the literature.^8,53,54^ Thus, in this work, it only needed to be established if the POMaC/PEGDA 3D printed materials hydrolytically degraded. Accelerated degradation using NaOH_(aq.)_ was performed to enable screening of the polymer degradation rates for the different 3D printed parts in a short period of time, as has been done previously in the literature.^8^ Cuboid specimens [7.5 mm × 7.5 mm × (3.9 ± 0.8) mm, *W* × *L* × *H*] were fabricated by molding of the different composition inks in a 12-well silicone chamber (Thistle Scientific, Glasgow, UK) under the UV LED of the Allevi 3 printer as per the conditions stated above in the description of the adhesive testing method. The specimens, which had been formed by molding, were then weighed and placed in pre-weighed 40 μm mesh cell strainers (for ease of handling), with the mass of the specimen *plus* strainer recorded as the starting mass (Starlab, Milton Keynes, UK) before being placed into an NaOH solution (1 M) and incubated at 37 °C for 72 h (corresponding to the point at which all samples had degraded). During incubation, the strainers containing the specimens were periodically removed, thoroughly dried, and the strainers containing the degrading specimens were weighed, before being placed back into the 1 M NaOH solution until 72 h. The mass difference of a cell strainer over 72 h in 1 M NaOH at 37 °C was determined to be ±1%. The mass difference of the specimens at the different time points was calculated by comparing the initial mass of the specimen (calculated by subtracting the mass of the strainer at *t* = 0 from the mass of specimen + strainer at *t* = 0) with the mass of the specimens at the different time points (calculated by subtracting the mass of the strainer + specimen at *t* = *X* from the mass of strainer at *t* = 0). Therefore, positive values of % mass change corresponds to uptake of 1 M NaOH solution within the specimen (resulting in swelling) and negative values of % mass change corresponds to the degradation of a part or the whole of a specimen—the hydrolysis degradation products remain solubilized within the 1 M NaOH solution. The results are presented as the means ± standard deviation.

### Biocompatibility Experiments

Cell permeable resazurin-based solution (PrestoBlue), which is used to quantitatively measure the cell proliferation, was used to determine the cell viability of fibroblast cells (3T3-L1, American Type Culture Collection (ATCC) no. CL-173) in presence of the varying ratio copolymers of POMaC/PEGDA. In brief, prior to the cell viability assessment, cells were seeded into a 96-well plate at a density of 2.5 × 10^4^ per well and supplied with 90 μL/well cell culture media [Dulbecco’s Modified Eagle Medium (DMEM) containing 10% heat-activated fetal bovine serum with 1% penicillin–streptomycin antibiotics (Pen-strep)] and L-glutamine and incubated overnight at 37 °C and with 5% CO_2_ incubator to allow cell adherence. On the consecutive day, the POMaC/PEGDA copolymer specimens of different wt % ratio compositions were washed three times with PBS to remove any excess uncross-linked inks or photoinitiator and then added into the wells and cultured for 48 h. To evaluate the cell viability of the 3T3-L1, 10 μL PrestoBlue was added to each well and incubated for 15 min at 37 °C. The absorbance of the reagent at 570 nm was measured using a Thermo Scientific Varioskan plate reader. Cells in absence of copolymer specimens and cell culture medium were used as controls. This experiment was repeated at least four times (*n* ≥ 4) and the specimens were 3D printed.

## Results and Discussion

A POMaC pre-polymer was synthesized through a co-condensation reaction of three different monomers (citric acid, maleic anhydride, and 1,8-octanediol) in a 1:4:5 M ratio at 140 °C for 2 h under a N_2_ atmosphere (Figure 2A). In contrast to the reported reaction conditions in the literature, in this work the reaction time for POMaC was reduced from 3 to 2 h, as it was qualitatively determined that the POMaC pre-polymer synthesized after 3 h was too viscous for molding and 3D printing.^8^ Furthermore, based on previous reports on POMaC, the 1:4:5 M ratio of reactants was chosen (referred to as “POMaC8” in the literature) to result in the highest number of double bonds within the pre-polymer chain to increase the photocross-linking rate during molding and 3D printing.^8^ After lyophilization, the resulting clear and colorless viscous product was characterized by ^1^H NMR and FT-IR spectroscopy (Figure 2B,C). The NMR data and the FT-IR data were consistent with the spectra reported in the literature^6,8^ and thus it was deduced that the desired POMaC pre-polymer product had been successfully synthesized. The POMaC pre-polymer was also characterized with MALDI-TOF mass spectrometry and the most intense peak (100% relative abundance) at *m*/*z* 546 g·mol^–1^ was attributed to the [M]^+•^ species, which corresponds to a pre-polymer oligomer of the structure shown in Figure 2A where R_1_ and R_2_ are −OH moieties (Figure S4). MALDI-TOF mass spectrometry was used for characterization of the POMaC pre-polymer because it was utilized in the first reported synthesis of POMaC by Tran et al.,^8^ and it has been utilized for characterization of other CABEs.^1,10,37,55,56^

After confirmation of the desired pre-polymer product, the biomaterial inks consisting of POMaC pre-polymer (100–Y) wt % combined with Y wt % PEGDA700 (where *Y* = 0, 5, 10, 20, 30, 40, or 50) and 5 wt % Irgacure 2959 photoinitiator (Table 1 and Figure 2D) were mixed. Due to the desirable properties highlighted in the introduction (vide supra), POMaC was the major wt % component in all the ink compositions, apart from the 50/50 ink, which was included to evaluate the effect of PEGDA on the properties of the 3D printed materials across the full wt % range in which POMaC is the major component. These biomaterial inks were then used for either molding or 3D printing.

For molding, silicone molds were used to allow for facile handling and all the biomaterial inks were successfully molded into cuboids of 7.5 mm × 7.5 mm x (3.9 ± 0.8) mm, using the UV LED source of the 3D printer. It was qualitatively determined that with increasing wt % of POMaC, longer UV curing times were required. Following the definition of effective energy as stated by Stowe^57^ and knowing that the “100% power” setting on the software interface corresponds to 20 mW·cm^–2^,^52^ the effective energy required for curing the 50/50 copolymer was calculated as 72 J·cm^–2^ for 1 h, and the effective energy required for curing the 100% POMaC was calculated as 1728 J·cm^–2^ over 24 h. Knowing that the area of the cuboid molds in this work was 0.5625 cm^2^, the total energies for curing the 50/50 polymer mold and the 100/0 polymer mold were 40.5 and 972 J, respectively. However, it must be considered that the average thickness of the molds in this work were (3.9 ± 0.8) mm, thus if the thickness difference is considered, the normalized energy for the 50/50 ink is ∼1 J and for the 100/0 ink is ∼24 J. The lower energy value for the 50/50 ink compared to pure POMaC in the work of Montgomery et al.^58^ is to be expected as the addition of PEGDA700 increases the number of photocross-linkable groups per unit volume and also increases the rate of cross-linking by decreasing the viscosity of the ink. While the energy value for the 100/0 ink is larger than the value reported in the literature,^58^ it is assumed that there is a uniform intensity of UV light through the molded piece when normalizing for the thickness difference, but this is not physically representative of the absorption of UV light, as described by the Beer–Lambert Law. Furthermore, it should be noted that Montgomery et al. also used a further thermal cross-linking treatment after the photocuring step,^58^ whereas in this work only photocuring was used. Nonetheless, these results demonstrated that the curing of POMaC-based inks is possible with the UV LED curing system of a desktop off-the-shelf 3D printer.

**Figure 3:**
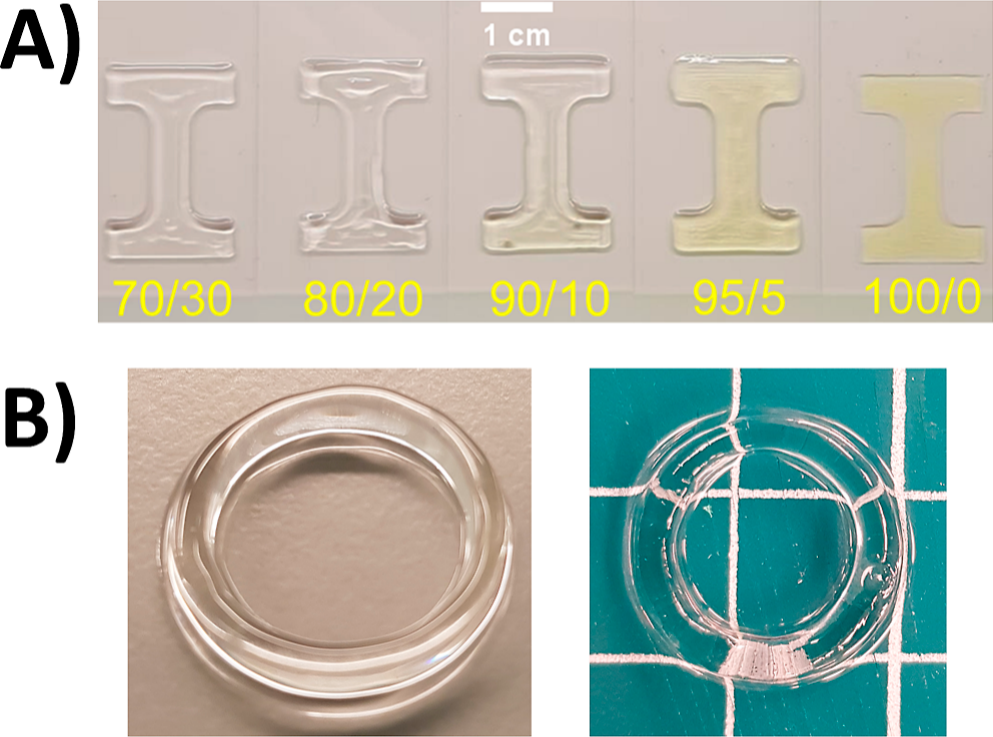
(A) Photographs of a selection of the 3D printed “dog bone” pieces, with the biomaterial ink composition indicated below each piece in yellow text, highlighting the high fidelity of the 3D printing of the .*stl* “dog bone” model. “Dog bone” pieces 95/5 and 100/0 in this figure were printed using 20 wt % photoinitiator. Scale bar = 1 cm. (B) Photographs of some of the 3D printed ring pieces (80/20, left and 70/30, right) further highlighting the 3D printing of complex shapes (white grid is equal to 1 cm).

The 3D printing of the different composition inks was then investigated. The inks were loaded into syringes fitted with 25-gauge Luer Lock dispensing tips and then placed into the Allevi CORE printhead. The desired *.stl* models, that had been designed using standard 3D design software, were uploaded to the Allevi web-based interface and an iterative printing process was performed. Both a “dog bone” *.stl* model and a ring *.stl* model were used (Figure S1). An iterative process of trial-and-error 3D printing, informed by the parameters needed to print the previous biomaterial ink composition, was used for each different biomaterial ink. The 3D printing parameters that were altered were extrusion pressure, print speed, UV intensity, and UV exposure times during and after printing. Finally, a set of parameters for each biomaterial ink composition, which resulted in 3D printing of the *.stl* models with good printability, assessed by qualitative visual assessment, was achieved (Table 2). Examples of the resulting 3D printed “dog bone” and ring pieces are shown in Figure 3A,B. However, it should be noted that the 95/5 and 100/0 “dog bone” pieces were only 3D printed to a qualitative visually acceptable printability using 20 wt % photoinitiator and when using the same printing parameters optimized for the 3D printing of 95/5 and 100/0 ring pieces.

During this process, several trends between printing parameters and the composition of the biomaterial inks were observed. For instance, there was a linear relationship between the required extrusion pressure and wt % of POMaC pre-polymer in the biomaterial ink and the resulting linear regression equation [extrusion pressure = 1.671(Weight % POMaC)—83.643] enables prediction of the required extrusion pressure required for inks with any wt % of POMaC between 50–100 wt % POMaC (Figure S5A). This can be explained by the viscosity of the biomaterial inks, as with increasing wt % of POMaC the viscosities were qualitatively observed to increase. The viscosity of PEGDA700 at room temperature is 98 mPa·s,^59^ whereas that of structurally similar poly(alkyl citrates) is known to be >210 mPa·s,^60^ thus the greater the wt % of POMaC, the more viscous the biomaterial ink and thus a greater extrusion pressure was required. Additionally, the required UV intensity during 3D printing generally increased with increased wt % of POMaC (Figure S5C). By decreasing the wt % of PEGDA700, the concentration of available double bonds available to undergo radical polymerization decreased, thus this increased both the UV curing time and the UV intensity required for sufficient cross-linking to polymerize in a suitable timescale for extrusion printing. However, it was determined that there was no clear relationship between the print nozzle movement speed and wt % POMaC (Figure S5B).

**Figure 4:**
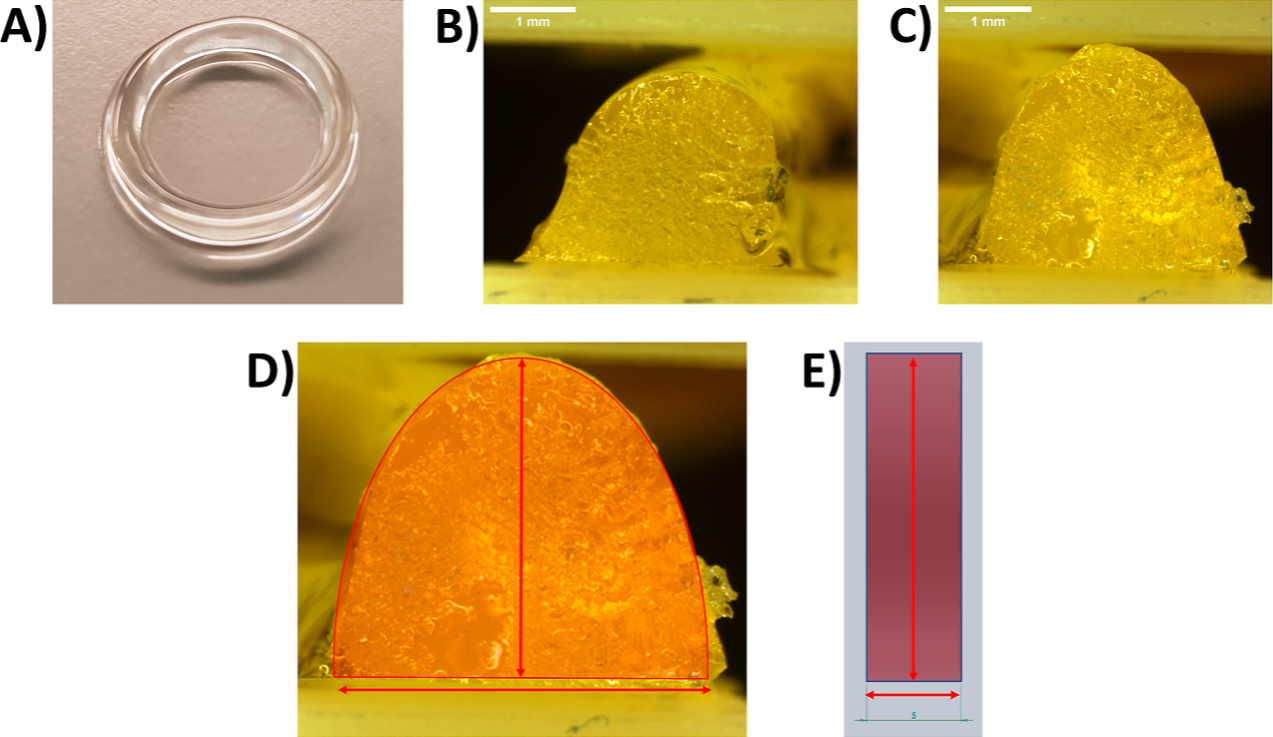
(A) Photograph of an 80/20 3D printed ring piece (B) Example microscopy image of the left-hand cross-sectional area of a 3D printed ring piece. Scale bar = 1 mm. (C) Example microscopy image of the right-hand cross-sectional area of a 3D printed ring piece. Scale bar = 1 mm. (D) Example microscopy image of a cross-sectional area with an overlaid schematic defining the measured cross-sectional area (red semi-transparent hemi-ellipse), the cross-sectional height (vertical red arrow), and the cross-sectional width (horizontal arrow). (E) Cross-sectional area of the ideal ring *.stl* model with an overlaid schematic defining the measured cross-sectional area (red semi-transparent rectangle), the height (vertical red arrow), and the width (horizontal arrow).

Then, the printability of the different biomaterial inks with different compositions was quantitatively assessed. The 3D printed ring pieces (Figures S1C,D and 4A) were halved with a scalpel so that microscopy images of the cross sections of each 3D printed ring (Figure 4B,C) could be captured, to enable the realized height, width, and area of each cross section (Figure 4D) to be measured using *Fiji* image analysis software,^51^ and compared to the design height, width, and area of the cross section of the ideal ring structure (.*stl* model) (Figure 4E).

The desired ideal height of the cross section of the ring was 3.75 mm, the desired ideal width of the cross section of the ring was 1.5 mm and thus the ideal cross-sectional area was 5.625 mm^2^. To quantify the printability, the ratio of the measured values for the 3D printed ring pieces to the ideal values are calculated, with a ratio of 1 corresponding to the measured value being equivalent to the ideal dimension value. The ratios between the ideal and measured dimensions (cross-sectional height, cross-sectional width, and cross-sectional area) are shown in Figure S6A-C. It can be noted that the ratios of the area, width, and height are different from the desired design value = 1. However, from all the plots, 70/30 had the best printability, that is, the values of the ratios of area, width, and height were closest to the design value. Therefore, with further optimization of the 70/30 composition, better printability is expected. This experiment implies that to achieve an ideal printability, a compensation factor should be considered.

**Figure 5:**
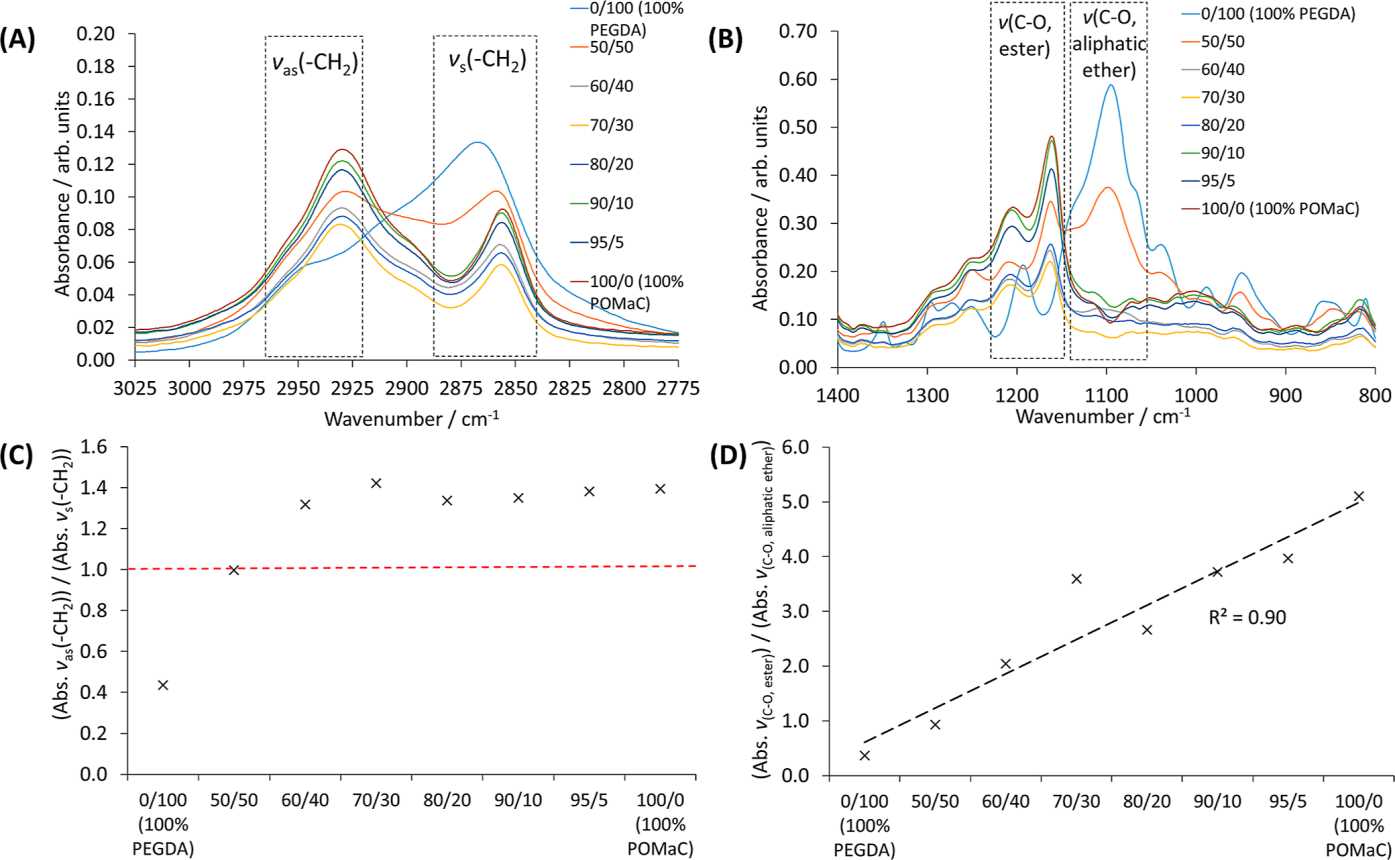
(A) Upon reduction of the PEGDA700 wt % content, the magnitude of the asymmetric methylene C–H stretch (ν_as_, ∼2930 cm^–1^) increased, whereas the symmetric methylene C–H stretch (ν_s_, ∼ 2855 cm^–1^) decreased, indicating a change in polymer chain packing density. (B) Upon reduction of the PEGDA700 wt % content, the magnitude of the C–O stretch of the ester functional groups (ν_C-O, ester_, ∼1160 cm^–1^) increased due to more ester groups, whereas the magnitude of the C–O stretch of the aliphatic ether groups (ν_C-O, aliphatic ether_, ∼1093 cm^–1^) decreased due to fewer aliphatic ether functional groups. (C) Ratio of the intensity of the asymmetric methylene C–H stretch (ν_as_, ∼2930 cm^–1^) to the intensity of the symmetric methylene C–H stretch (ν_s_, ∼2855 cm^–1^) increased with decreasing PEGDA700 wt % content until PEGDA700 ≤ 40 wt % whereupon the ratio remained a constant value. The red line is a guide to the reader indicating an intensity ratio value of 1.0. (D) Ratio of the intensity of the C–O stretch of the ester functional groups (ν_C–O, ester_, ∼1160 cm^–1^) to the intensity of the C–O stretch of the aliphatic ether groups (ν_C–O, aliphatic ether_, ∼1093 cm^–1^) increased linearly with decreasing PEGDA700 wt % content due to fewer ethylene glycol repeating units present and a subsequent increase in the number of ester linkages (see polymer structures in Figure 1).

The 3D printed pieces were then characterized with FT-IR (Figures S7, 5A–D, and S8) and Raman spectroscopy (Figure S9), and TGA (Figure S10 and Table 3). There were clear differences in the FT-IR spectra of the copolymer 3D printed pieces with decreasing PEGDA700 wt % content (Figure S7); for example, in the C–H stretch region (approx. 3000–2800 cm^–1^), which corresponds to methylene C–H asymmetric (ν_as_) and symmetric (ν_s_) stretches (Figure 5A) and in the fingerprint region between approx. 1200–1000 cm^–1^, which corresponds to C–O stretches of both ester and aliphatic ether functional groups (Figure 5B).

**Table 3:** TGA of the Copolymer Pieces 3D Printed with the Different Biomaterial Inks^a^

composition	*T*_*o*_/°C	*T*_*p*_/°C	total mass loss/%	est. water content/%
0–100 (100% PEGDA)	171	357	98.5	0.2
50/50	190	397	97.8	0.5
60/40	176	407	98.6	0.7
70/30	177	389	99.6	0.9
80/20	177	392	99.3	0.9
90/10	167	381	99.2	0.8
95/5	161	395	98.6	0.6
100/0 (100% POMaC)	163	395	98.7	0.6

a There were few differences between the different compositions, apart from onset temperature, *T*_*o*_, which decreased with increasing POMaC content. The water mass content for all compositions was less than <1%. *T*_*o*_ is defined as per ASTM-E2550-17.^62^

The FT-IR spectra of the 3D printed pieces confirm that upon reduction of the PEGDA700 wt % content the intensity of the asymmetric methylene C–H stretch (ν_as_) peak increased, whereas the intensity of the symmetric methylene C–H stretch (ν_s_) peak decreased (Figure 5A). This was further confirmed by comparison of the intensity of the ν_as_ peak to the intensity of the ν_s_ peak for the different biomaterial ink compositions as shown in Figure 5C. It was also concluded that the ν_as_/ν_s_ ratio increases when the PEGDA700 wt % content decreases—up to PEGDA700 ≤ 40 wt % whereas it remained at a constant value of ∼1.4. An increase in the magnitude of the ν_as_/ν_s_ ratio can indicate that there are larger changes in the dipole moments of the vibrations due to an increase in free volume allowing more space for polymer segmental motions.^61^ An increase in free volume can be attributed to the increased number of side chains due to the citric acid unit in the polymer chain of POMaC (see Figure 1) upon decreasing PEGDA700 wt %, which results in poorer packing of the polymers chains and thus affords an increase in free volume until PEGDA700 ≤ 40 wt %. In addition, the intensity of the C–O stretch of the ester functional groups (ν_C–O, ester_) increases with decreasing PEGDA700 wt % content, whereas the intensity of the C–O stretch of aliphatic ether groups (ν_C-O, aliphatic ether_) decreased (Figure 5B), as confirmed by the linear increase in the ratio of the intensities of the C–O stretch peaks (ν_C–O, ester_/ν_C–O, aliphatic ether_) in Figure 5D. This is attributed to fewer ethylene glycol repeating units (repeating unit of PEGDA, see Figure 1) in the 3D printed copolymers with decreasing PEGDA700 wt % content and increase in the number of ester functional groups, thus confirming the difference in wt % content of both polymers in each 3D printed copolymer piece. Finally, it was determined that for each copolymer piece consisting of the different 3D printed biomaterial ink compositions that there were still unpolymerized C=C double bonds present due to the presence of the double bond (C=C) stretch peak (ν_C=C_, ∼1643 cm^–1^) in the FT-IR spectra of the pieces (Figure S7), but it is noted that the presence of remaining unpolymerized C=C double bonds in POMaC-based polymers has been reported in the literature.^8^ Upon further investigation of the ratio of the intensity of the ν_C=C_ peak to the intensity of the carbonyl (C=O) stretch peak (ν_C=O_, ∼1724 cm^–1^) there was no clear difference between the different biomaterial ink compositions (Figure S8), but the presence of remaining C=C bonds was confirmed. Finally, analysis of the Raman spectra revealed that there was an increase in the carbonyl C=O Raman shift (Δṽ_C=O_) with decreasing PEGDA700 wt % with a resulting increase in POMaC wt % content, which has a greater number of carbonyl functional groups (Figure S9). Thus, the Raman spectral results further confirm the difference in wt % content of both polymers in each 3D printed copolymer piece.

The 3D printed pieces were also characterized with TGA (Figure S10 and Table 3). For all biomaterial ink compositions, there was near complete mass loss after heating to 800 °C and the estimated water mass content, but there was no apparent trend between the different compositions and the peak temperature (*T*_p_). However, there was a trend of decreasing onset temperature (*T*_o_), as defined as per ASTM E2550-17,^62^ with increasing POMaC wt %. This can be attributed to poorer packing between the polymer chains with increasing wt % of POMaC due to a greater number of side chains from the citric acid moieties. Finally, the water content for all biomaterial compositions was <1 wt %, which is corroborated by the hydrophobic nature of POMaC pre-polymer, as noted during the synthesis (vide supra) and in the literature.^58^ In the case of the low water content of 0/100 (100% PEGDA), neat PEGDA was used instead of forming a PEGDA hydrogel, as is typically described in the literature.^63^

**Figure 6:**
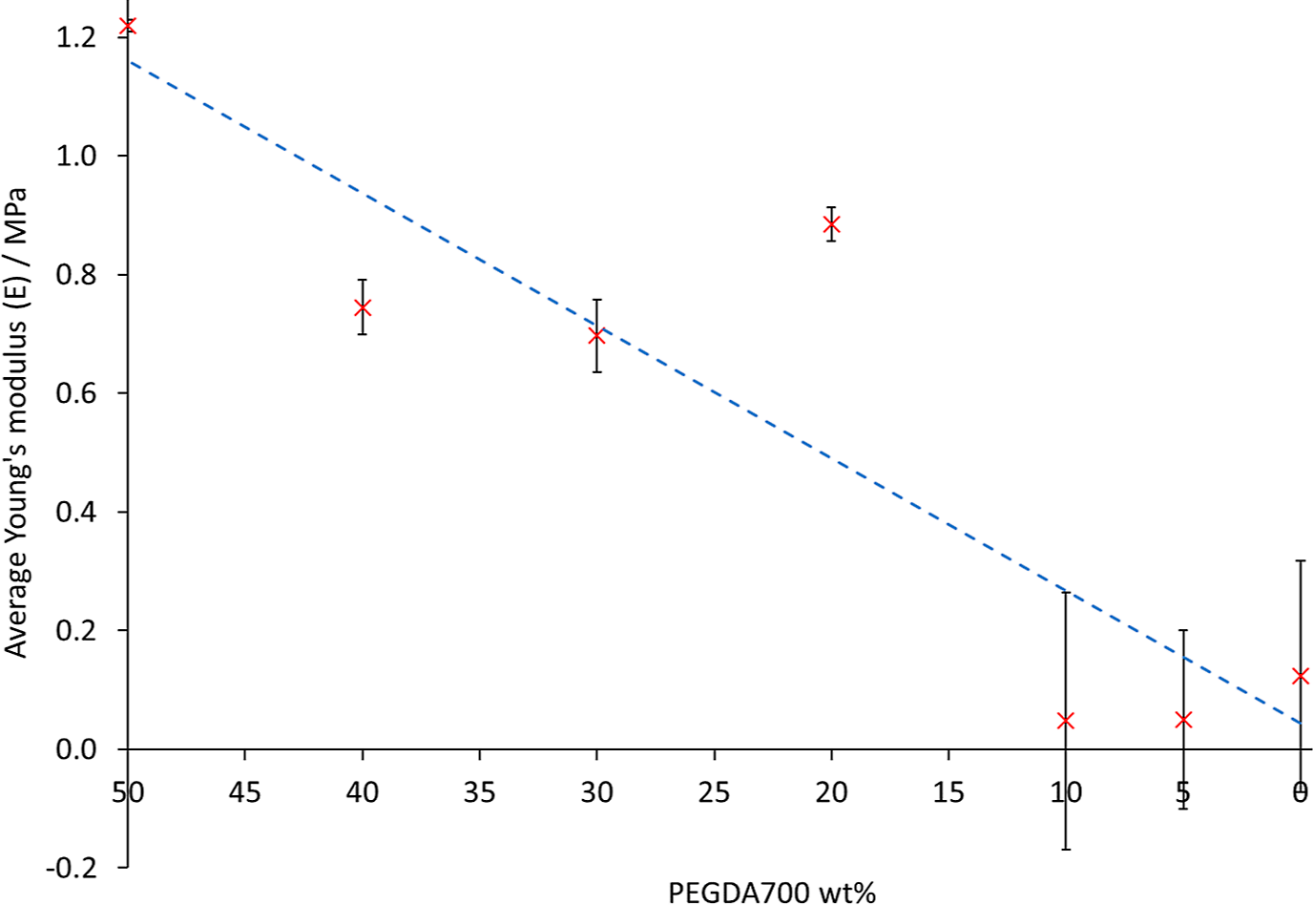
With decreasing PEGDA700 wt %, there is a decrease in the Young’s moduli (*E*), which is attributed to a decrease in cross-link density with decreasing PEGDA700 wt %. The dashed blue line is a guide for the eye only.

For potential utilization of these 3D printed materials for wound sealing or wearable biomedical technologies, the mechanical and adhesive properties need to be measured and understood. Therefore, the mechanical properties of 3D printed ring pieces of each biomaterial ink composition were evaluated through tensile stress–strain experiments as per the experimental setup in Figure S2. Displacement in the tension force direction was applied at a constant rate, and at each displacement value (in mm) the adhesive peak load values (in N) were recorded until the ring piece was observed to break. The data were then processed as per the formulae defined in ISO-37 to calculate average values of tensile strength at break (TS_b_/MPa) and elongation % at break (*E*_b_/%) for each biomaterial ink composition.^50^ The cross-sectional areas measured with optical microscopy during the printability evaluation experiments (vide supra) were used to plot the true stress–elongation % curves from which average Young’s modulus values (*E*) were determined. The average values of TS_b_, *E*_b_, and *E* for each biomaterial ink composition are given in Table 4 and the relationship between the average Young’s modulus (*E*) values and PEGDA700 wt % is shown in Figure 6.

**Table 4:** Average Values of Young’s Modulus (*E*), Tensile Strength at Break (TS_b_), and Elongation at Break (*E*_b_) Obtained through Mechanical Property Analysis of 3D Printed Ring Pieces Consisting of the Different Biomaterial Ink Compositions^a^

composition	avg. Young’s modulus, *E*/MPa	avg. tensile strength at break,TS_b_/MPa	avg. elongation at break, *E*_b_/%
50/50	1.22 ± 0.01	0.163 ± 0.010	15.44 ± 0.05
60/40	0.75 ± 0.05	0.093 ± 0.046	21.06 ± 9.60
70/30	0.70 ± 0.06	0.080 ± 0.101	18.34 ± 2.08
80/20	0.89 ± 0.03	0.103 ± 0.008	32.98 ± 8.49
90/10	0.05 ± 0.22	0.007 ± 0.010	30.57 ± 6.46
95/5	0.05 ± 0.15	0.016 ± 0.016	55.90 ± 5.44
100/0 (100% POMaC)	0.12 ± 0.19	0.030 ± 0.034	44.83 ± 1.62

a Average values of TS_b_ and *E*_b_ are defined as per ISO-37.^50^ Values are mean ± standard deviation.

There was no clear trend observed between the ink compositions and *E*_b_ or TS_b_ values, which was attributed to intra-variability in terms of the difference of ratios between the ideal and measured dimensions (the ideal design values = 1) for different specimens consisting of the same ink composition. Nonetheless, despite no clear linear regression relationship between average *E* values and PEGDA700 wt %, there is an overall decrease of E with decreasing PEGDA700 wt %. This is attributed to fewer cross-links with decreased PEGDA700 wt %. In addition, it is highlighted that the values of E for all the POMaC/PEGDA inks were in the region of E values for human tissues^64^ and in the region of typical *E* values for hydrogels in the literature,^65^ which further suggests the suitability of 3D printed POMaC/PEGDA copolymer for biomedical applications.

**Figure 7:**
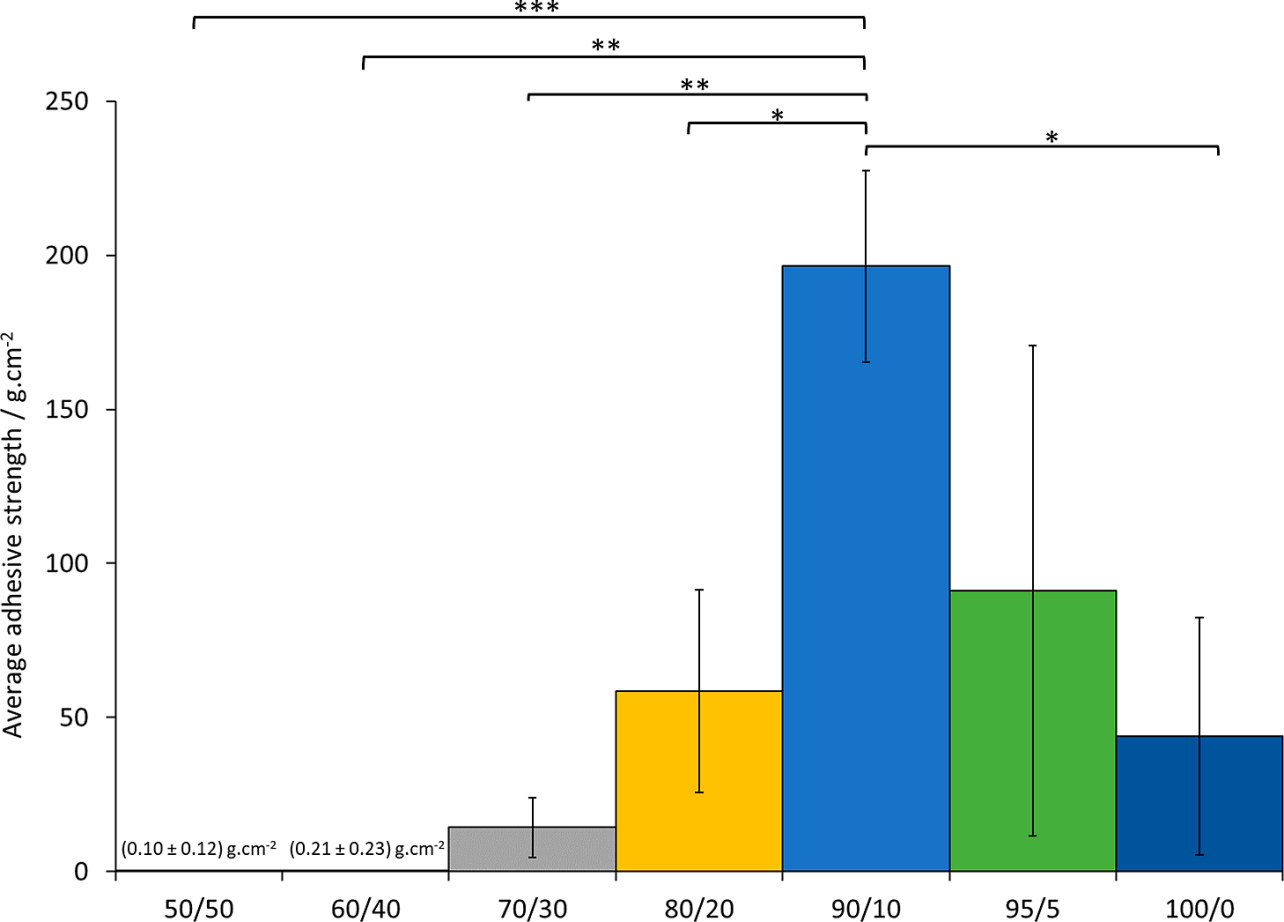
Average adhesive strengths measured, using the pull-off tensile adhesion test method, for each different biomaterial ink composition cured material, showing that the 90/10 composition was the “stickiest”, that is, has the highest average adhesive strength. The results are presented as the mean ± standard deviation, *n* ≥ 3. Statistical significance was evaluated using one-way ANOVA followed by post-hoc *t*-tests with the Holm–Bonferroni correction applied (* = *P* ≤ 0.05; ** = *P* ≤ 0.01; *** = *P* ≤ 0.001; otherwise not significant). The magnitude of the values and error bars for both the 50/50 and 60/40 compositions are too small to be visible on this plot and thus the values are indicated on the plot.

Then, the adhesive strength of specimens of each biomaterial ink composition was evaluated using the pull-off tensile adhesion test method. The highest magnitude value registered on the force gauge readout screen (Figure S3) before pull-off, due to adhesive failure and not cohesive failure, of a specimen from the bottom stainless-steel dolly was recorded and an average adhesive strength for each composition was calculated (*n* ≥ 3) (Figure 7).

As shown in Figure 7, the largest magnitude average adhesive strength was measured for the 90/10 biomaterial ink composition specimen—four times larger in magnitude than the average adhesive strength for the 100/0 “100% POMaC” biomaterial ink composition specimen. Indeed, there was a high statistically significant difference between the average adhesive strength of the 90/10 and 50/50 biomaterial ink compositions, very statistically significant differences between 90/10 and 60/40 and between 90/10 and 70/30, and a statistically significant difference between 90/10 and 80/20. More crucially, there was a statistically significant difference between 90/10 and 100/0, thus the 90/10 copolymers were on average stickier than pure POMaC 100/0. It is proposed that the enhanced adhesive strength of 90/10 compared to the other copolymers is due to inclusion of an optimal wt % of hydrophilic PEGDA to the relatively hydrophobic POMaC. Indeed, it is known in the literature that PEGDA is hydrophilic,^66^ and it has been successfully used as a co-monomer for enhancement of adhesive strength in other polymer systems.^67,68^ The lack of a statistically significant difference between 95/5 and 100/0 is attributed to the large error caused by poor cohesive strength of the 95/5 copolymer samples; adhesive failure without cohesive failure was difficult to achieve. Nonetheless, these results suggest that another benefit of copolymers of POMaC and PEGDA is tunable, and in some cases, increased adhesive strengths are achieved compared to pure POMaC (100/0).

**Figure 8:**
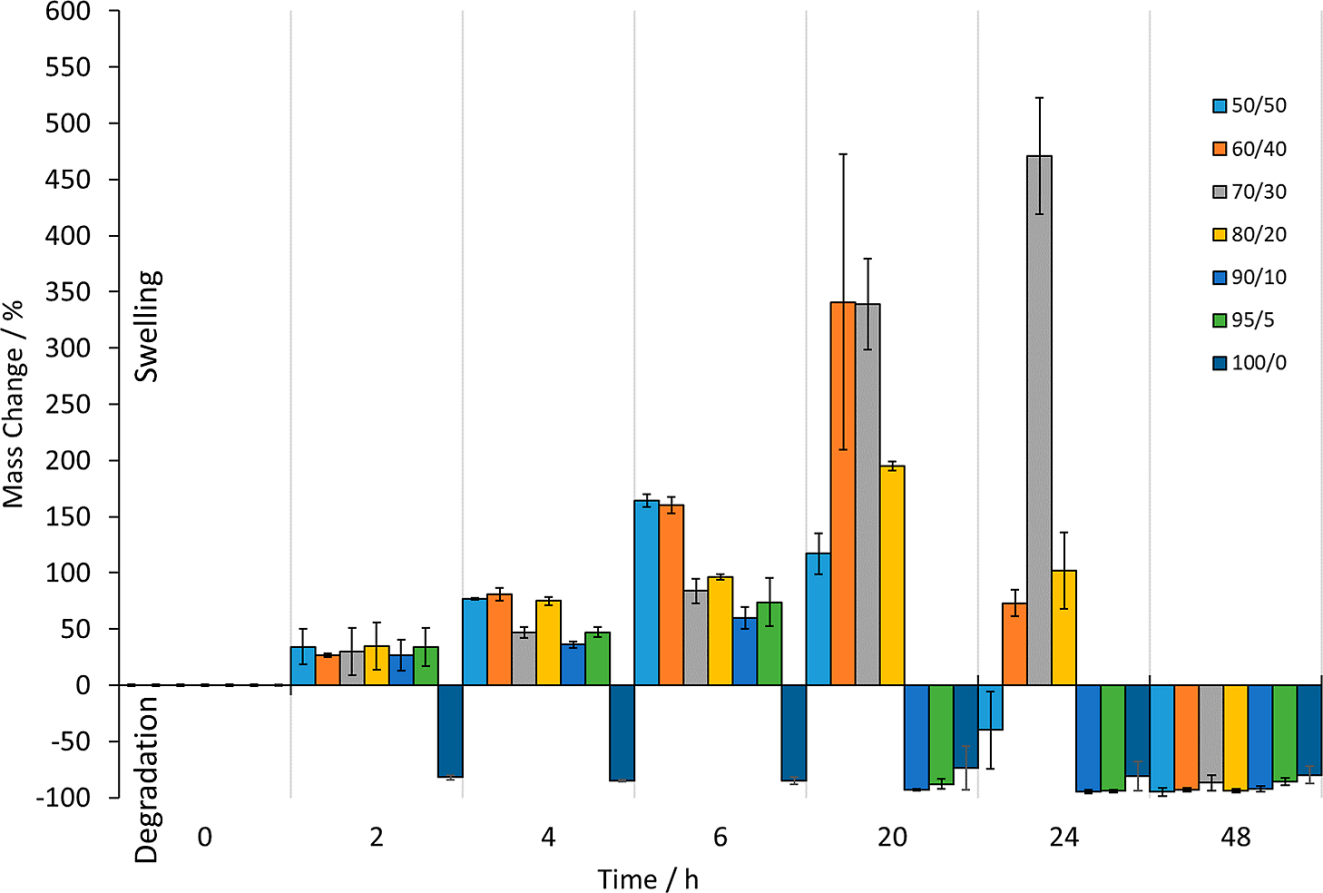
Degradation assay showing the mass changes recorded upon treatment of cured pieces of the different biomaterial ink composition materials with 1 M sodium hydroxide solution over a period of 48 h—degradation was accelerated through pragmatic use of 1 M sodium hydroxide solution.^8^ After 48 h, all pieces had fully degraded by visual observation. The results are presented as the mean ± standard deviation.

For potential biomedical applications, ideally the materials need to degrade so that the 3D printed material or a part is not permanently stuck on or within the body, which can cause further complications. It is known that POMaC is degraded under hydrolysis conditions and is biodegradable and that PEGDA is bioinert. Indeed, after 2 weeks, approximately 50% mass loss occurs for POMaC in PBS, and 80% mass loss degradation occurs in 10 weeks.^8^ In addition, the degradation rate of PEGDA-based materials in PBS can be tuned from hours to weeks,^53^ and significant in vivo degradation of PEGDA-based materials has been shown to occur within 12 weeks.^54^ Therefore, for this work, all that was required was determination of whether these composite materials, which contain varying amounts of degradable POMaC and degradable PEGDA, also degraded (Figure 8).

The cured pieces of the different biomaterial ink compositions were treated under accelerated degradation conditions (1 M sodium hydroxide solution at 37 °C with gentle agitation)—a pragmatic choice in terms of experiment time for determination of hydrolytic degradation.^8^ At first, all of the pieces were visually observed to swell, which was also confirmed for all compositions by the recorded mass changes, apart from the 100/0 biomaterial ink composition material, which fully degraded by visual observation within 2 h under these conditions (and confirmed by the mass change recorded). After 48 h, all the pieces were observed visually to have degraded, and this was further confirmed with the mass change results as all the cured biomaterial ink composition pieces underwent an approximate −100% mass change. However, before 48 h, the biomaterial ink composition that was most resistant to swelling and subsequent degradation was the 70/30 composition. These results allow for design of the composition of the POMaC/PEGDA biomaterial inks so that degradation time of the 3D printed pieces can be controlled.

**Figure 9:**
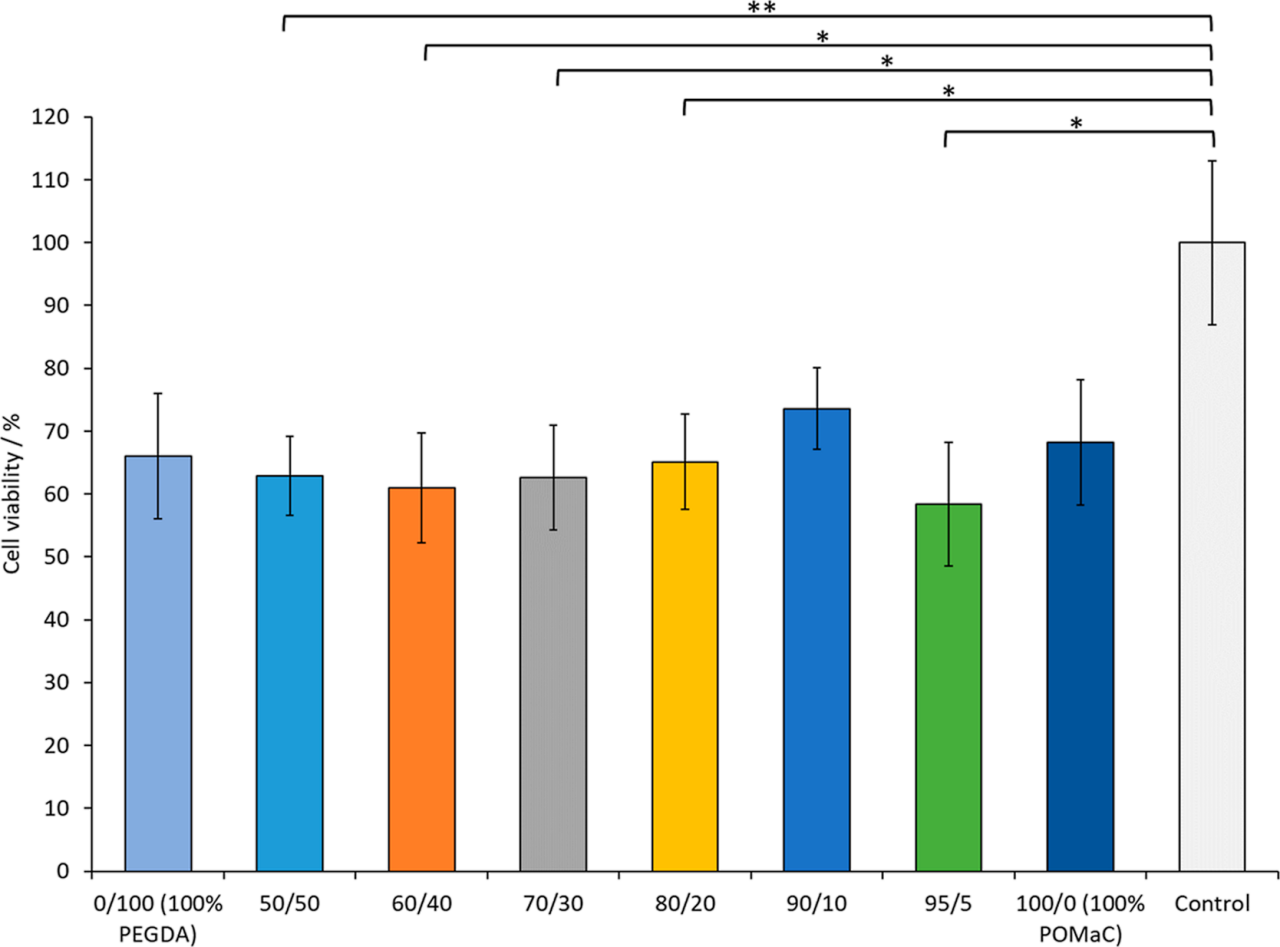
Cell viability assay: reduction of PrestoBlue reagent as a correlate of cell viability over a period of 48 h is plotted for different biomaterial ink compositions. Values are normalized to the control and shown as mean ± standard deviation, *n* ≥ 4. Statistical significance was evaluated using one-way ANOVA followed by post-hoc *t*-tests with the Holm–Bonferroni correction applied (* = *P* ≤ 0.05; ** = *P* ≤ 0.01; *** = *P* ≤ 0.001; otherwise not significant).

After confirming that the different biomaterial copolymer pieces were degradable, the biocompatibility was investigated. The cytotoxicity of the copolymer materials was evaluated using the resazurin-based PrestoBlue reagent. Metabolically active cells reduce the oxidized non-fluorescent blue resazurin to the red fluorescent dye resorufin by the mitochondria in live cells, a colorimetric change that is a quantifiable indicator of viability of cells in culture. The amount of resorufin is directly proportional to the number of viable cells. As depicted in Figure 8, a minimum of 60% viability was measured for all materials relative to the control, which highlights that these copolymer materials are suitable for biomedical applications. Furthermore, there are no statistically significant differences between the different copolymer compositions and 0/100 (100% PEGDA) and 100/0 (100% POMaC), thus the combinations of PEGDA with POMaC at different compositional ratios have little to no toxicity. However, the statistically significant difference in viability of 50/50, 60/40, 70/30, 80/20, and 95/5 relative to the control for all materials is attributed to the toxicity of residual Irgacure 2959 photoinitiator directly. Despite the known low toxicity of Irgacure 2959, ≥5 wt % photoinitiator was required for successful 3D printing of these biomaterial copolymers. Therefore, it is hypothesized that residual Irgacure 2959, not consumed during the photocross-linking step of 3D printing, may generate radical species that are toxic to the cells, thus resulting in a minimum of 60% cell viability for all copolymer compositions. However, it is predicted that the biocompatibility can be improved further as it has been shown previously that POMaC-based materials shows good biocompatibility up to and beyond 8 weeks in vivo (Figure 9).^69^

Overall, this proposed method, through modification of the ink formulations, affords a more generalizable approach to achieve 3D printing of elastomers with a wide range of mechanical and physicochemical properties, without the need to alter the chemical structure of the elastomer, nor use of a support material^39^ or fillers.

## Conclusions

This work is the first example of 3D printing of POMaC and POMaC-based copolymer biomaterials. It was determined that extrusion 3D printing of the POMaC-based inks could be optimized for, and achieved with, an off-the-shelf lab-based extrusion 3D bioprinter and the use of PEGDA as a copolymer. Qualitative assessment of the printed parts demonstrated that complex shapes could be achieved; however, quantitative assessment revealed that printing fidelity of these POMaC-based copolymer biomaterial inks needs further optimization. Nonetheless, it was also determined that the use of PEGDA does not impact the degradability or biocompatibility, compared to 100/0 (100% POMaC), while allowing for a significant increase in adhesive strength (90/10). Overall, it was determined that the use of PEGDA has two benefits: 3D printing of complex-shaped POMaC-based materials is achievable and the physical properties of the resulting 3D printed parts can be tuned, optimized, and altered through altering the ratio. Therefore, this work represents a significant advance toward the application of 3D printed POMaC-based inks for the next generation of personalized biomedical devices.

## Supporting Information Available

Ring and “dog bone”.*stl* file images; screenshot of mechanical property testing, experimental testing video, and representative true stress versus elongation curves; screenshot of adhesive experiment video and adhesive experiment schematic; MALDI mass spectrum; printing parameter trend plots; printability analysis plots; FT-IR spectra of 3D printed parts; plot of IR Abs. ν_C=C_/IR Abs. ν_C=O_ values; plot of Δṽ_C=O_ Raman shift values; and TGA curves (PDF)

## Notes

Raw data available at Imperial College London Research Computing Service data repository: http://doi.org/10.14469/hpc/10643.
