# Supplementary material for: 3D Printability Assessment of Poly(octamethylene maleate (anhydride) citrate) and Poly(ethylene glycol) Diacrylate Copolymers for Biomedical Applications
Source: ACS Appl Polym Mater. 2022 Jul 7;4(8):5457–70. doi: 10.1021/acsapm.2c00531 (PMC9379906; doi:10.1021/acsapm.2c00531)
Supplement: Supplementary file 1 — ap2c00531_si_001.pdf [file ap2c00531_si_001.pdf]

Supporting Information

3D printability assessment of poly(octamethylene maleate (anhydride) citrate) and poly(ethylene glycol) diacrylate copolymers for biomedical application

Dominic J. Wales†*[1], Meysam Keshavarz†[1], Carmel Howe[2] and Eric Yeatman*[3]

[1]Hamlyn Centre, Institute of Global Health Innovation, Imperial College London, London, SW7 2AZ, United Kingdom

[2]Department of Bioengineering, Imperial College London, London, SW7 2AZ, United Kingdom

[3]Department of Electrical and Electronic Engineering, Imperial College London, London, SW7 2AZ, United Kingdom

† = joint first authorship

*= corresponding authors: d.wales@imperial.ac.uk; e.yeatman@imperial.ac.uk

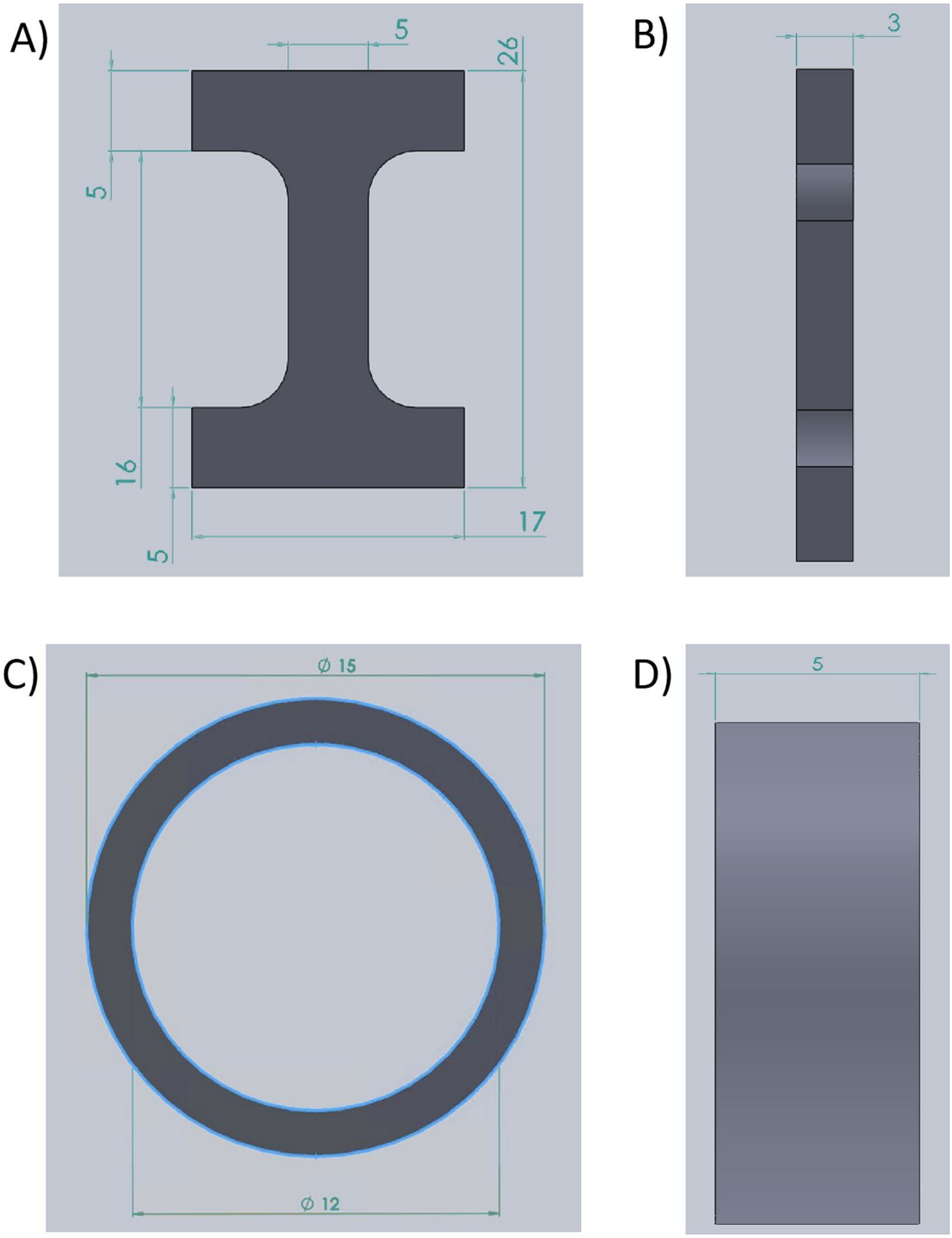

Figure S1: (A) Top view of the "dog bone" *.stl* model for 3D printing with measurements shown. **(B)** Side view of the "dog bone" *.stl* model for 3D printing with thickness measurement shown. **(C)** Top view of the ring *.st*l model for 3D printing with outer and inner diameter measurements shown. **(D)** Side view of the ring *.stl* model for 3D printing with thickness measurement shown. All measurements shown are in millimetres. The *.stl* designs were defined using 3D design software (SolidWorks, Dassault Systèmes, France).

A)

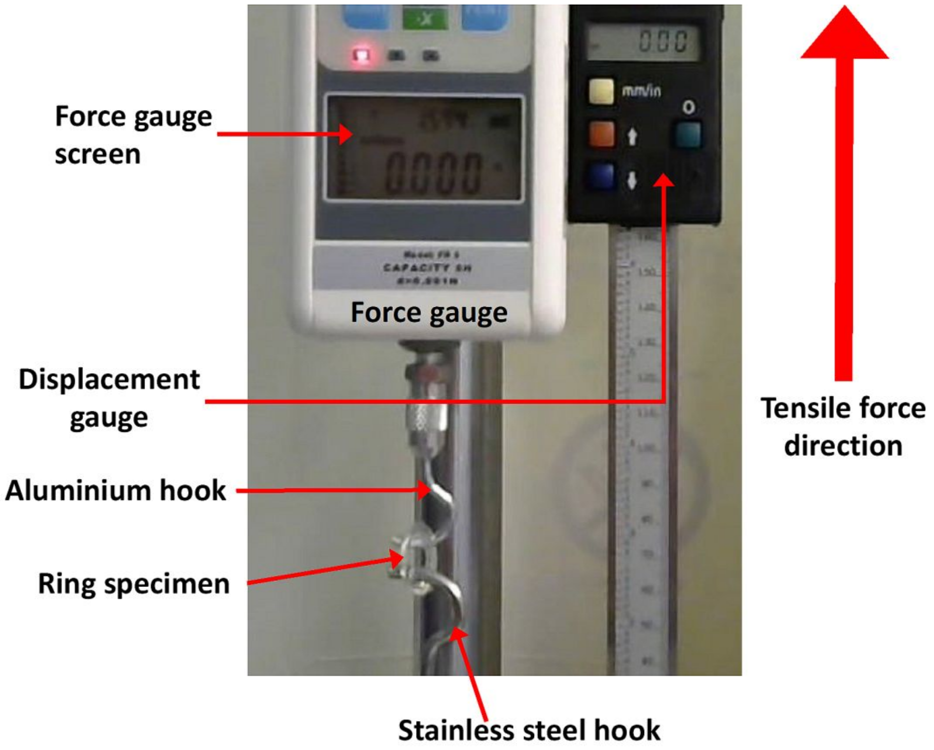

B)

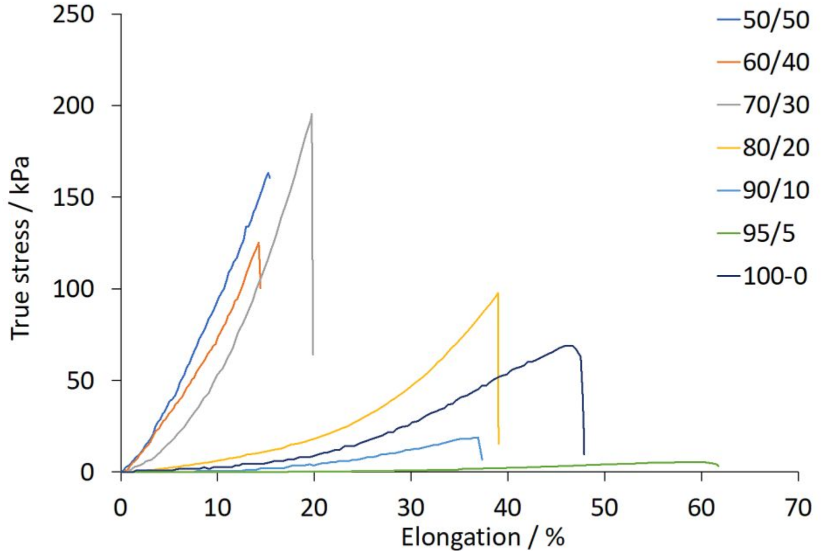

Figure S2: (**A**) Screenshot from a video recorded during a tensile stress-strain experiment showing the experimental set-up. The force loads and displacement values were extracted manually from the videos by transcribing the values registered on the readout screens from both the force gauge readout screen and the displacement gauge screen. (**B**) Representative example true stress – elongation curves for the different biomaterial ink compositions.

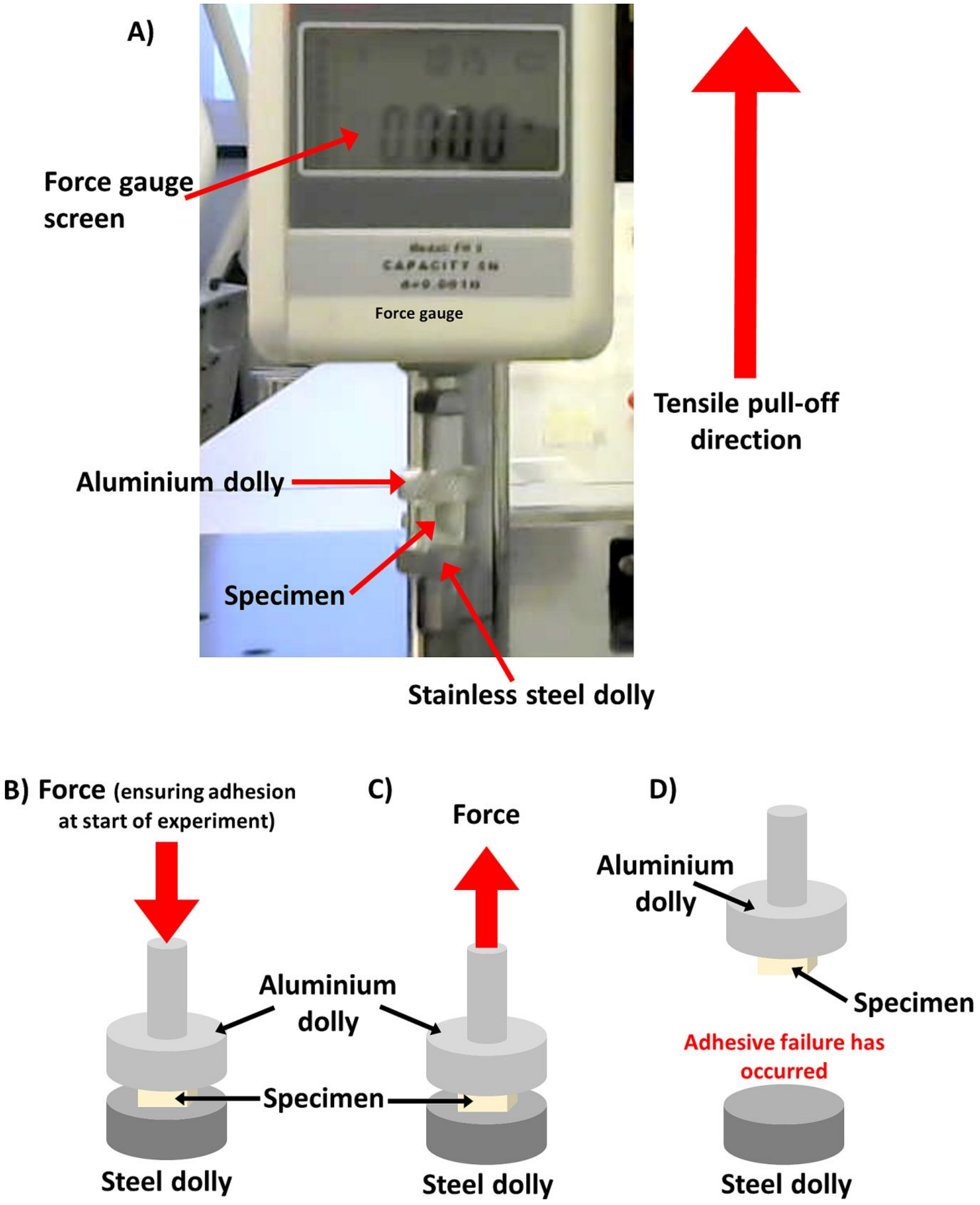

Figure S3:(**A**) A screenshot from an experimental video recorded during a pull-off tensile adhesive test. The adhesive peak loads were extracted manually from the videos by transcribing the highest magnitude value registered on the readout screen from the force gauge readout screen before adhesive failure, "pull-off" of the specimen from the stainless-steel dolly was achieved. (**B**) Schematic showing the start of the experiment whereupon a specimen was placed between the aluminium and steel dollies and force (direction indicated by the red arrow) was applied to ensure adhesion. (**C**) Schematic of during the experiment, where upon force was applied (direction indicated by the red arrow) until (**D**) adhesive failure, "pull-off" had occurred (schematic).

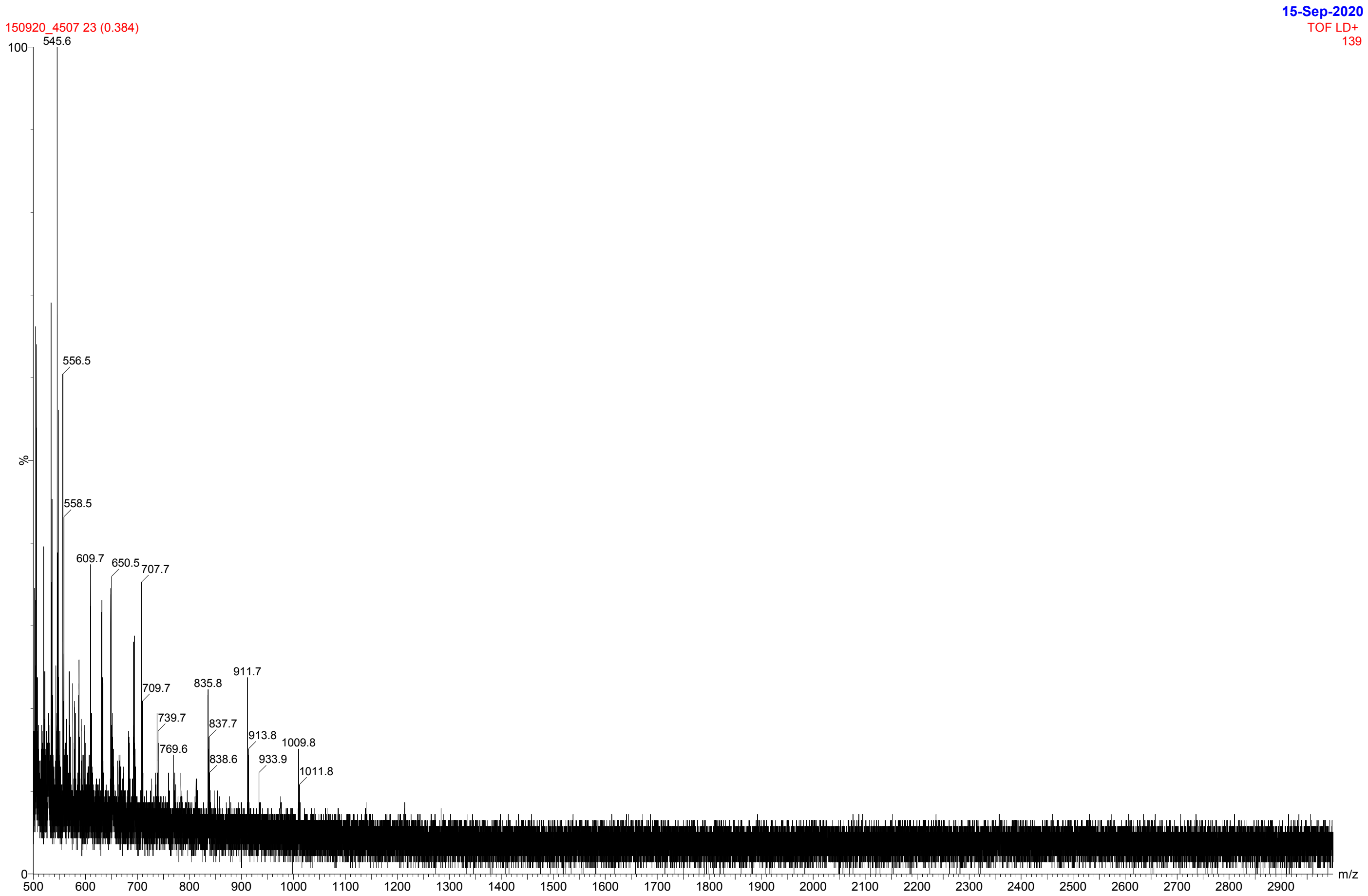

Figure S4: MALDI-TOF mass spectrum of POMaC pre-polymer oligomer. The most intense peak (100% relative abundance) at m/z 546 g.mol^{-1} was attributed to the [M]+• species, which corresponds to a pre-polymer oligomer of the structure shown in **Figure 2A** when R_1 and R_2 are -OH moieties

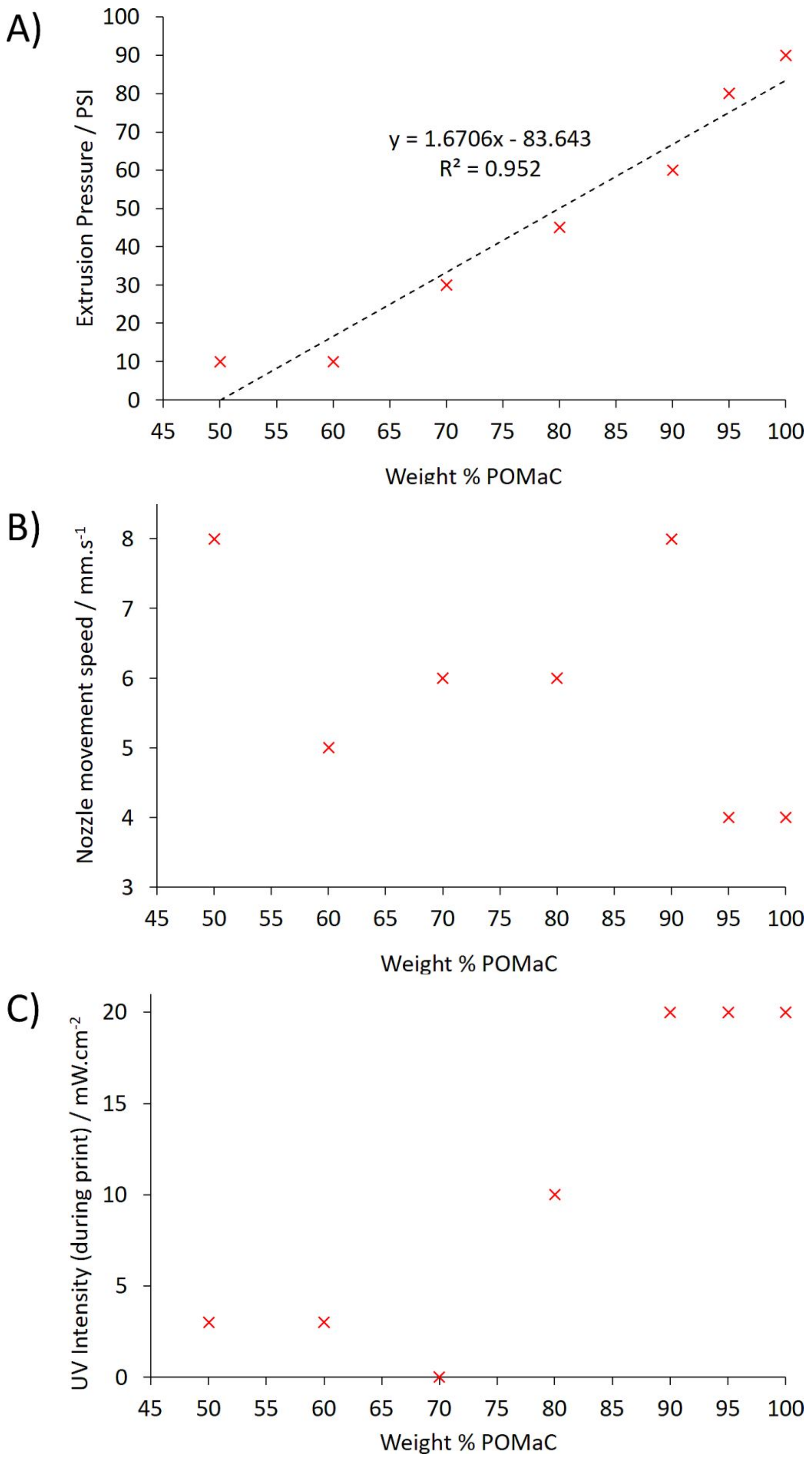

Figure S5:(A) With increasing POMaC wt% a greater extrusion pressure was required to achieve 3D printing of the biomaterial inks. The extrusion pressure values for the biomaterial ink compositions 95/5 and 100/0 were obtained during the ring printing optimisation process using 5 wt% photoinitiator. The dashed line is the linear regression with equation *Extrusion pressure = 1.6706(Weight% POMaC) – 83.643* that can be used to determine the extrusion pressure required for any POMaC : PEGDA ink in the weight % POMaC range from 50 – 100 wt%. (**B**) There was no clear relationship between printing nozzle movement speed and weight % POMaC. (**C**) For increased weight % POMaC higher UV intensity during 3D printing was required.

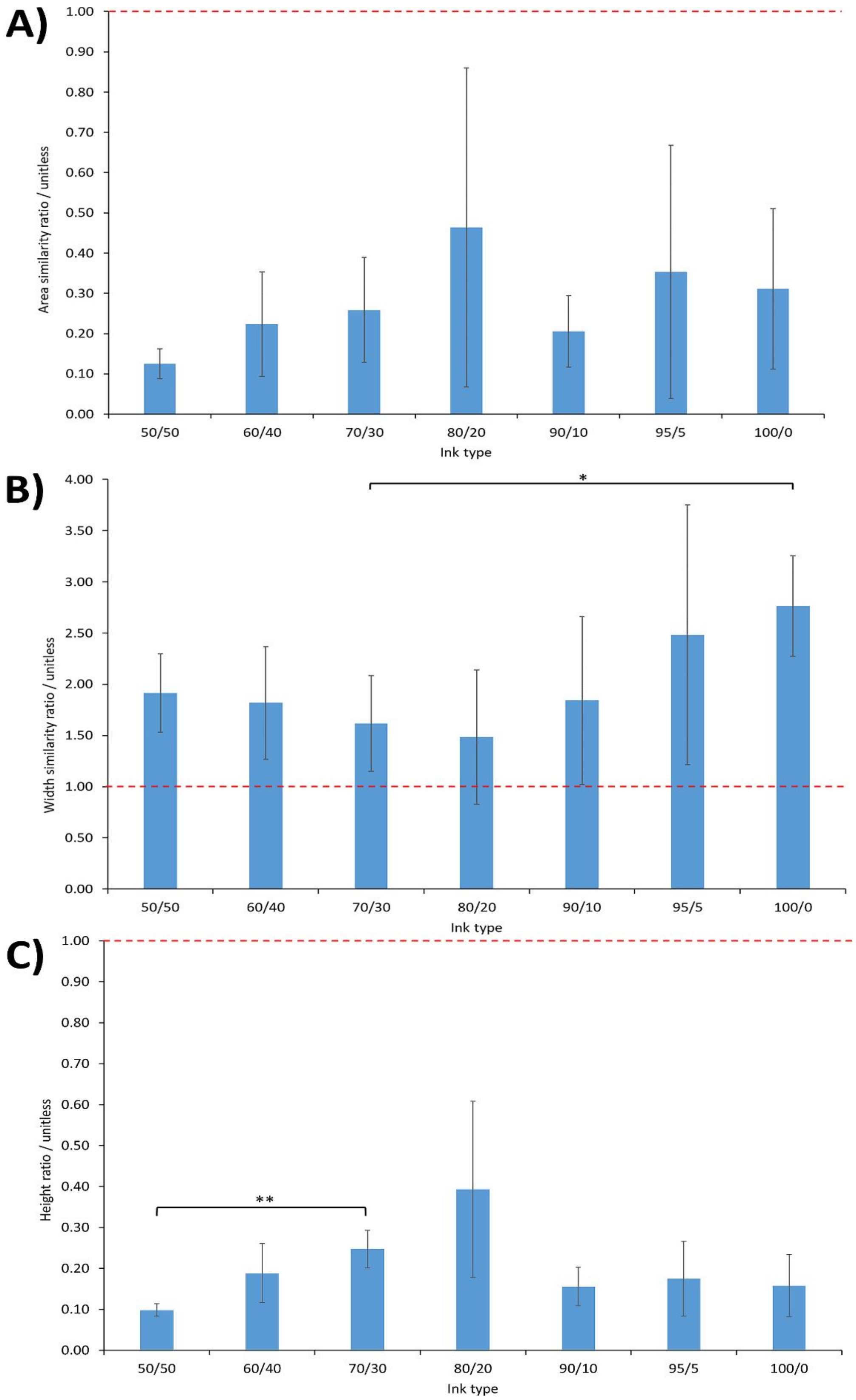

Figure S6: (A) For all biomaterial ink compositions the area similarity ratios were smaller in magnitude than the ideal value = 1 (denoted by the dashed red line), i.e., the cross-sectional areas of all the printed rings was smaller than the desired cross sectional area as defined in the *.stl* model. **(B)** For all biomaterial ink compositions, the width similarity ratios were larger in magnitude than the ideal value = 1 (denoted by the dashed red line), i.e., the widths of the cross sections of all the printed rings was larger than the desired cross-sectional width as defined in the *.stl* model. (**C**) However, all height similarity ratios were smaller in magnitude than the ideal value = 1(indicated by the red dashed line). Error bars correspond to the mean ± standard deviation. $N \geq 4$. Statistical significance was evaluated using one-way ANOVA followed by post-hoc t-tests with the Holm-Bonferroni correction applied (* = $P \leq 0.05$; ** = $P \leq 0.01$; *** = $P \leq 0.001$; otherwise not significant).

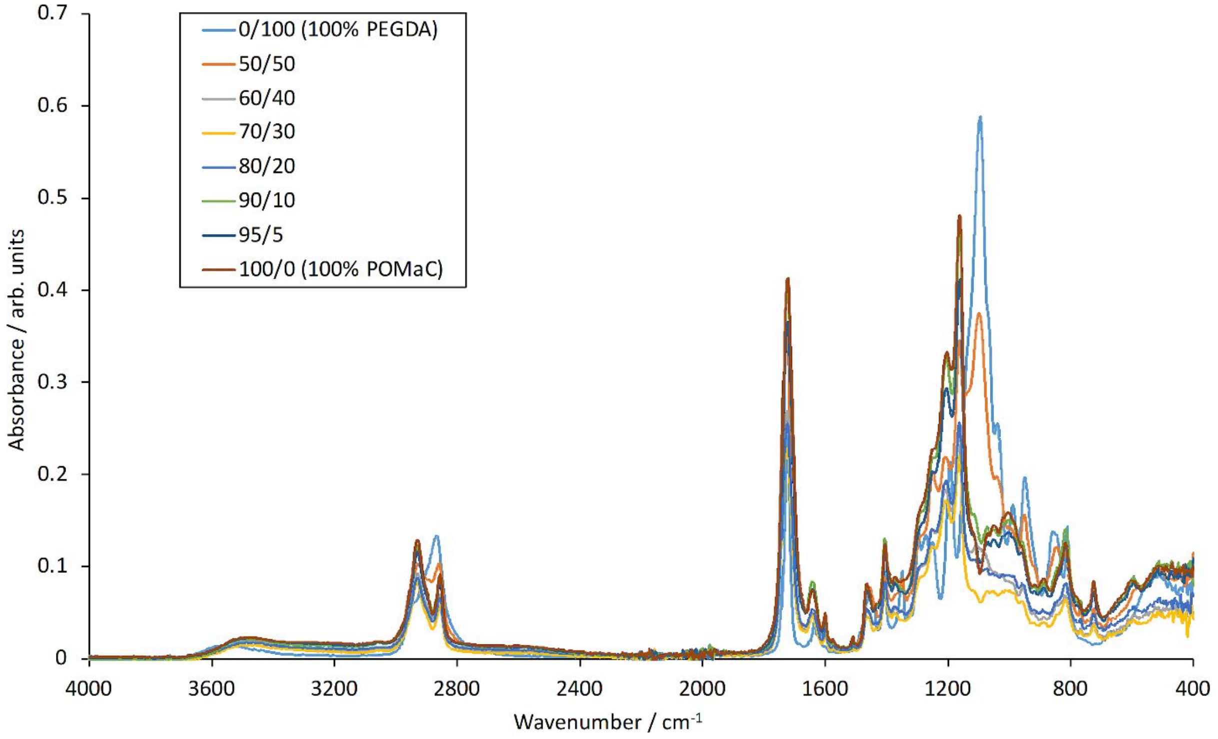

Figure S7: The FT-IR spectra of the printed pieces demonstrated significant differences in the fingerprint region and the C-H alkyl stretch region (approx. 2800-3000 cm^{-1}) between the spectra with decreasing PEGDA wt% content.

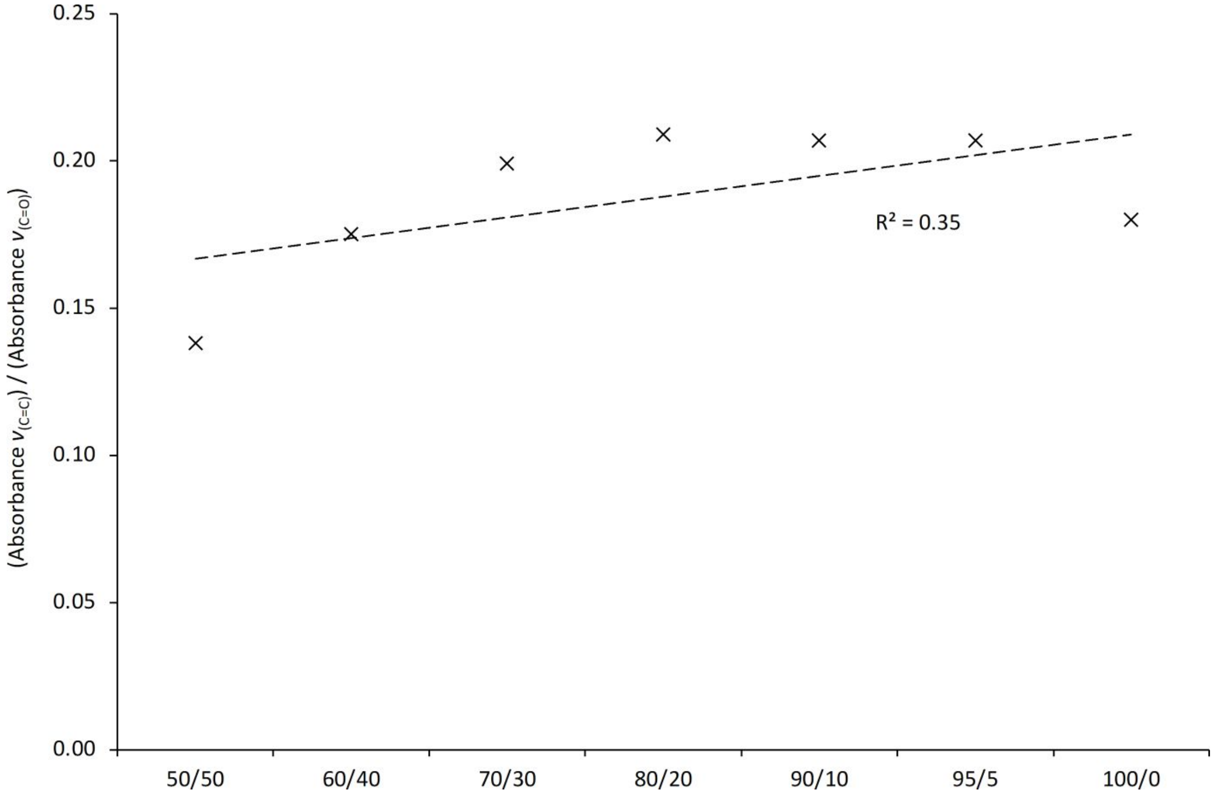

Figure S8: There was no clear difference in the ratios of the intensity of the $\nu_{C=C}$ (~1643 cm^{-1}) peak to the intensity of the $\nu_{C=O}$ (~1724 cm^{-1}) for each different biomaterial ink composition.

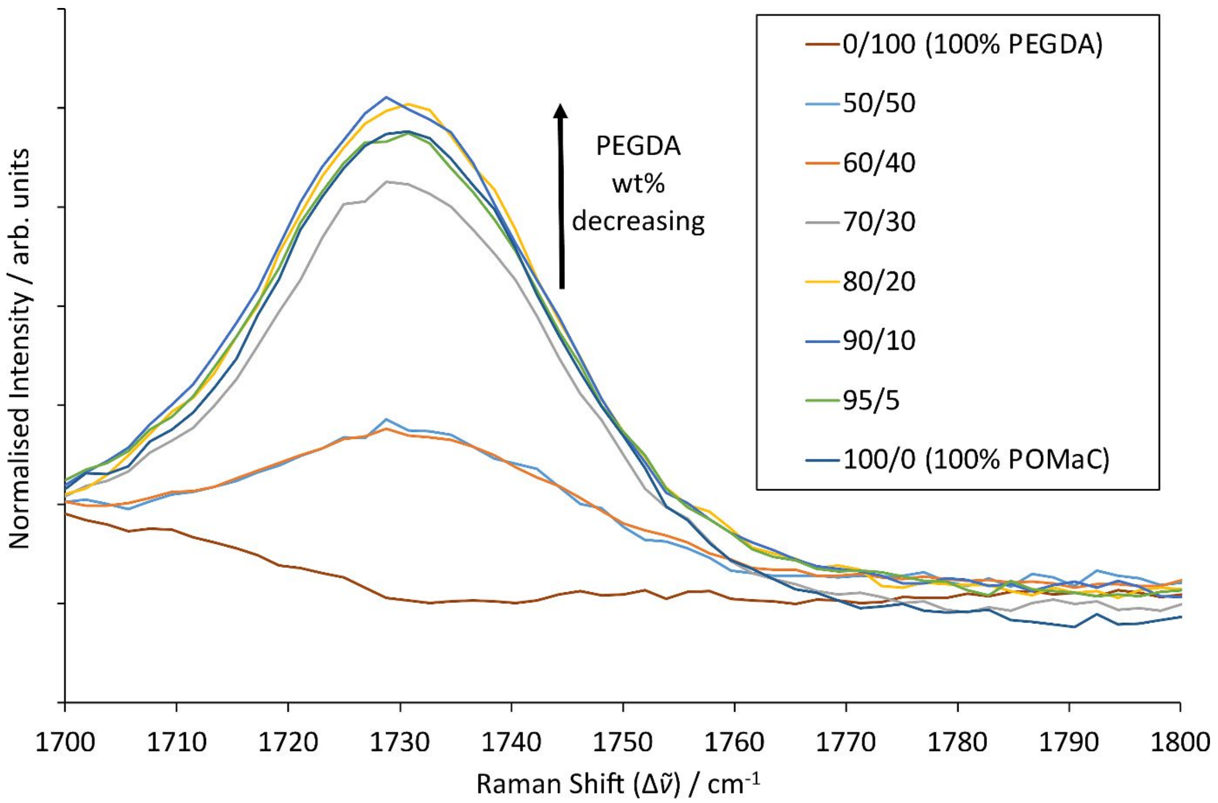

Figure S9: Increasing intensity of the Raman shift attributed to carbonyl functional groups ($\Delta\tilde{\nu}_{C=O} \approx 1730$ cm^{-1}) due to decreasing PEGDA700 wt% thus leading to higher wt% content of POMaC that contains a greater number of carbonyl functional groups.

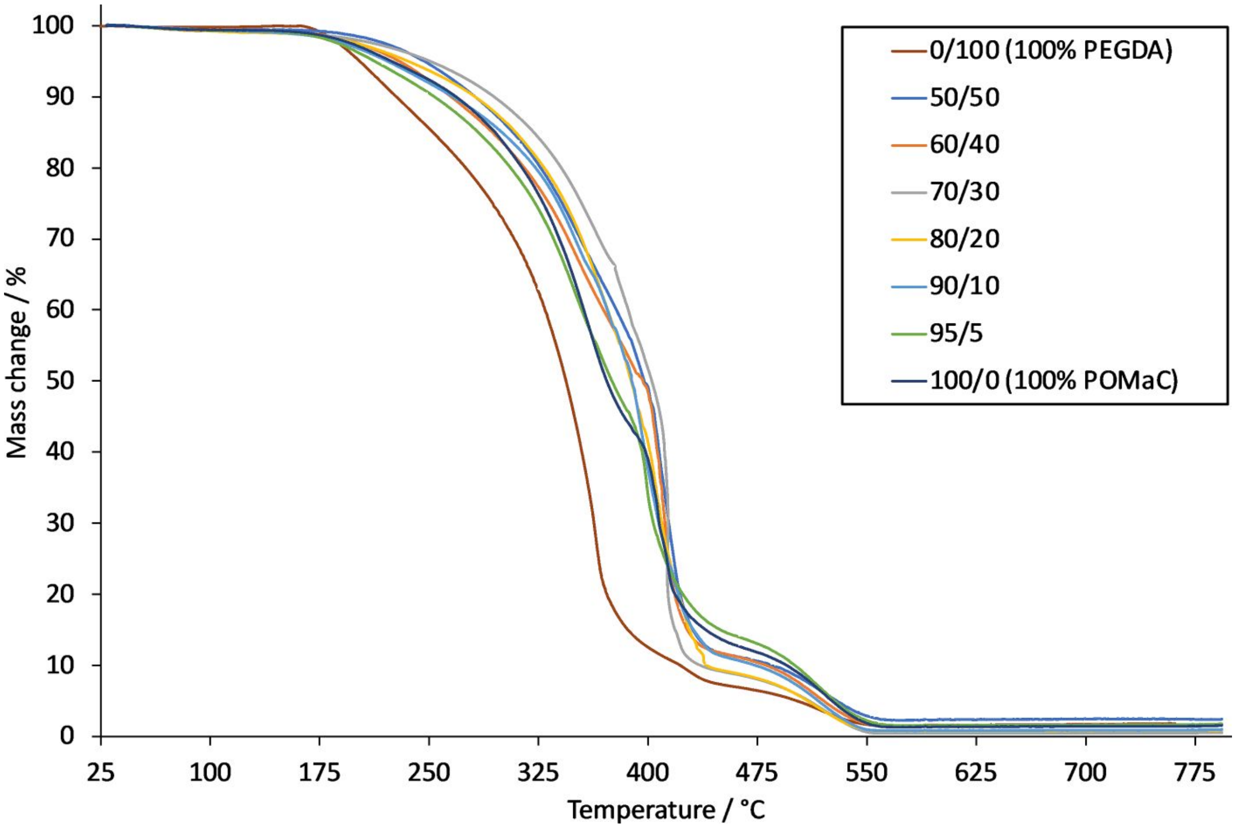

Figure S10: Thermal gravimetric analysis curves for the different 3D printed pieces.
